# Homozygous loss-of-function mutations in *MNS1* cause laterality defects and likely male infertility

**DOI:** 10.1371/journal.pgen.1007602

**Published:** 2018-08-27

**Authors:** Asaf Ta-Shma, Rim Hjeij, Zeev Perles, Gerard W. Dougherty, Ibrahim Abu Zahira, Stef J. F. Letteboer, Dinu Antony, Alaa Darwish, Dorus A. Mans, Sabrina Spittler, Christine Edelbusch, Sandra Cindrić, Tabea Nöthe-Menchen, Heike Olbrich, Friederike Stuhlmann, Isabella Aprea, Petra Pennekamp, Niki T. Loges, Oded Breuer, Avraham Shaag, Azaria J. J. T. Rein, Elif Yilmaz Gulec, Alper Gezdirici, Revital Abitbul, Nael Elias, Israel Amirav, Miriam Schmidts, Ronald Roepman, Orly Elpeleg, Heymut Omran

**Affiliations:** 1 Department of Pediatric Cardiology, Hadassah, Hebrew University Medical Center, Jerusalem, Israel; 2 Monique and Jacques Roboh Department of Genetic Research, Hadassah, Hebrew University Medical Center, Jerusalem, Israel; 3 Department of General Pediatrics, University Hospital Muenster, Muenster, Germany; 4 Department of Human Genetics, Radboud University Medical Center, Nijmegen, the Netherlands; 5 Radboud Institute for Molecular Life Sciences, Radboud University Nijmegen, Nijmegen, the Netherlands; 6 Pediatric Genetics Division, Center for Pediatrics and Adolescent Medicine, Faculty of Medicine, Freiburg University, Freiburg, Germany; 7 Pediatric Pulmonology Unit, Hadassah-Hebrew University Medical Center, Jerusalem, Israel; 8 University of Health Sciences, Kanuni Sultan Suleyman, Training and Research Hospital, Department of Medical Genetics, Istanbul, Turkey; 9 Pediatric Department, Ziv Medical Center, Faculty of Medicine, Bar Ilan University, Safed, Israel; 10 Saint Vincent Hospital, Nazareth, Faculty of Medicine, Bar Ilan University, Israel; 11 Department of Pediatrics, University of Alberta, Edmonton, Alberta, Canada; 12 Pediatric Pulmonology Unit, Tel Aviv Medical Center, Tel Aviv, Israel; Monash University, AUSTRALIA

## Abstract

The clinical spectrum of ciliopathies affecting motile cilia spans impaired mucociliary clearance in the respiratory system, laterality defects including heart malformations, infertility and hydrocephalus. Using linkage analysis and whole exome sequencing, we identified two recessive loss-of-function *MNS1* mutations in five individuals from four consanguineous families: 1) a homozygous nonsense mutation p.Arg242* in four males with laterality defects and infertility and 2) a homozygous nonsense mutation p.Gln203* in one female with laterality defects and recurrent respiratory infections additionally carrying homozygous mutations in *DNAH5*. Consistent with the laterality defects observed in these individuals, we found *Mns1* to be expressed in mouse embryonic ventral node. Immunofluorescence analysis further revealed that MNS1 localizes to the axonemes of respiratory cilia as well as sperm flagella in human. In-depth ultrastructural analyses confirmed a subtle outer dynein arm (ODA) defect in the axonemes of respiratory epithelial cells resembling findings reported in *Mns1*-deficient mice. Ultrastructural analyses in the female carrying combined mutations in *MNS1* and *DNAH5* indicated a role for MNS1 in the process of ODA docking (ODA-DC) in the distal respiratory axonemes. Furthermore, co-immunoprecipitation and yeast two hybrid analyses demonstrated that MNS1 dimerizes and interacts with the ODA docking complex component CCDC114. Overall, we demonstrate that MNS1 deficiency in humans causes laterality defects (*situs inversus*) and likely male infertility and that MNS1 plays a role in the ODA-DC assembly.

## Author summary

Although no clear explanation is yet provided for the correct establishment of the left-right body asymmetry in human, animal studies have clearly shown that tiny-hair-like organelles in the ventral node of the embryo—called motile nodal monocilia—beat regularly and play an important role in this process. To date, different genetic variants were identified to cause laterality defects but the list is still incomplete. Here, we describe two loss-of-function variants in *MNS1* in individuals suffering from laterality defects and infertility. This finding is particularly important because laterality defects can also be associated with increased risk of congenital heart disease. By examining a specific case with loss-of-function variants in *MNS1* and *DNAH5* (the latter encodes for DNAH5, a main component of the motor protein complexes providing the ciliary beating), we show that MNS1 plays a role in stabilizing structures in the motile cilium that helps the attachment of motor proteins–such as DNAH5- to the microtubules to ensure a correct beating of the cilia. Further studies of the function of MNS1 may implicate new biological pathways affecting susceptibility to laterality defects and infertility.

## Introduction

Cilia assemble on most cell types of the human body to perform diverse biological roles [1]. Non-motile primary cilia function in mechano- and chemosensation as well as in photoreception and olfaction, in addition to an essential role in several signal transduction pathways (noncanonical Wnt and Hedgehog pathways) [2]. Motile cilia and flagella exhibit several tissue and cell-type specific functions.

Establishment of the left-right body axis in vertebrates is an evolutionarily conserved process for which in mammals the embryonic node plays an essential role. A rotational leftward flow established by rotational movement of motile cilia is thought to result in transport of signaling molecules to the correct side of the embryo where this signal defines the establishment of the left-right body axes [3]. Analyses of mouse mutants affected by impaired left-right body axes’ development have proven that dysmotility or abnormal differentiation of nodal monocilia correlate directly with laterality defects [4]. Further, laterality defects can also be observed as a consequence of defective signal reception at the embryonic node and/or defects within the left-right patterning signaling pathways itself such as NODAL, Bmp or FGF signaling [3]. Situs abnormalities include *situs inversus totalis* (mirror-image reversal), left/right isomerism or *situs ambiguous* also known as heterotaxy. If heterotaxy occurs, congenital heart disease (CHD) can be frequently observed [5–6].

The underlying genetic defects associated with randomization of body laterality in human are therefore heterogeneous, including motile- as well as non-motile ciliary causes and non-ciliary causes. To date, human mutations have been identified in a number of genes including *LEFTY1* (MIM 603037), *LEFTY2* (MIM 601877) [7], *ACVR2B* (MIM 602730) [8], *CFC1* (MIM 605194) [9], *NEK8* (MIM 609779) [10], *INV* (MIM 243305) [11] and *CFAP53* (*CCDC11* (MIM 614759) [12–13].

However, laterality defects often occur (>50%) in individuals harboring mutations in genes which are necessary for the proper structure and function of the motile cilia—resulting in a condition clinically known as Primary Ciliary Dyskinesia (PCD; MIM# 244400) due to defective mucociliary clearance in the respiratory system [14]. PCD associated with chronic sinusitis, bronchiectasis and *situs inversus* is also described as Kartagener’s syndrome (KS) [15]. Male infertility can also be associated with PCD resulting from dysmotile/immotile sperm flagella. Mutations in the majority of the genes cause a combined phenotype comprising randomization of left/right body asymmetry, male infertility and defective ciliary clearance of the airways.

MNS1, a meiosis specific nuclear structural 1 protein, has been identified in the proteomes of human bronchial epithelium [16]. More recently, *Mns1*^*-/-*^ mice have been reported that exhibit: i) randomization of left/right asymmetry and laterality defects, ii) male infertility with sperm immotility and sperm flagellar defects, and iii) partial defects of outer dynein arms (ODAs: motor proteins providing the mechanical force for ciliary movement) in tracheal cilia [17].

In this study, through the collaboration of three centers in Germany, Israel and The Netherlands, we analysed 85 individuals with laterality defects but not classical PCD and identified by whole exome sequencing identical loss-of-function (LOF) mutations in *MNS1* (NM_018365) in four individuals from three unrelated families. Additionally, we identified a second *MNS1* homozygous mutation in a KS affected individual through linkage analysis and homozygosity mapping. Sanger sequencing of 134 PCD-affected individuals with or without laterality defects did not reveal any additional mutations in *MNS1*.

## Results

### Identification of *MNS1* mutations through whole exome sequencing

**Fig 1:**
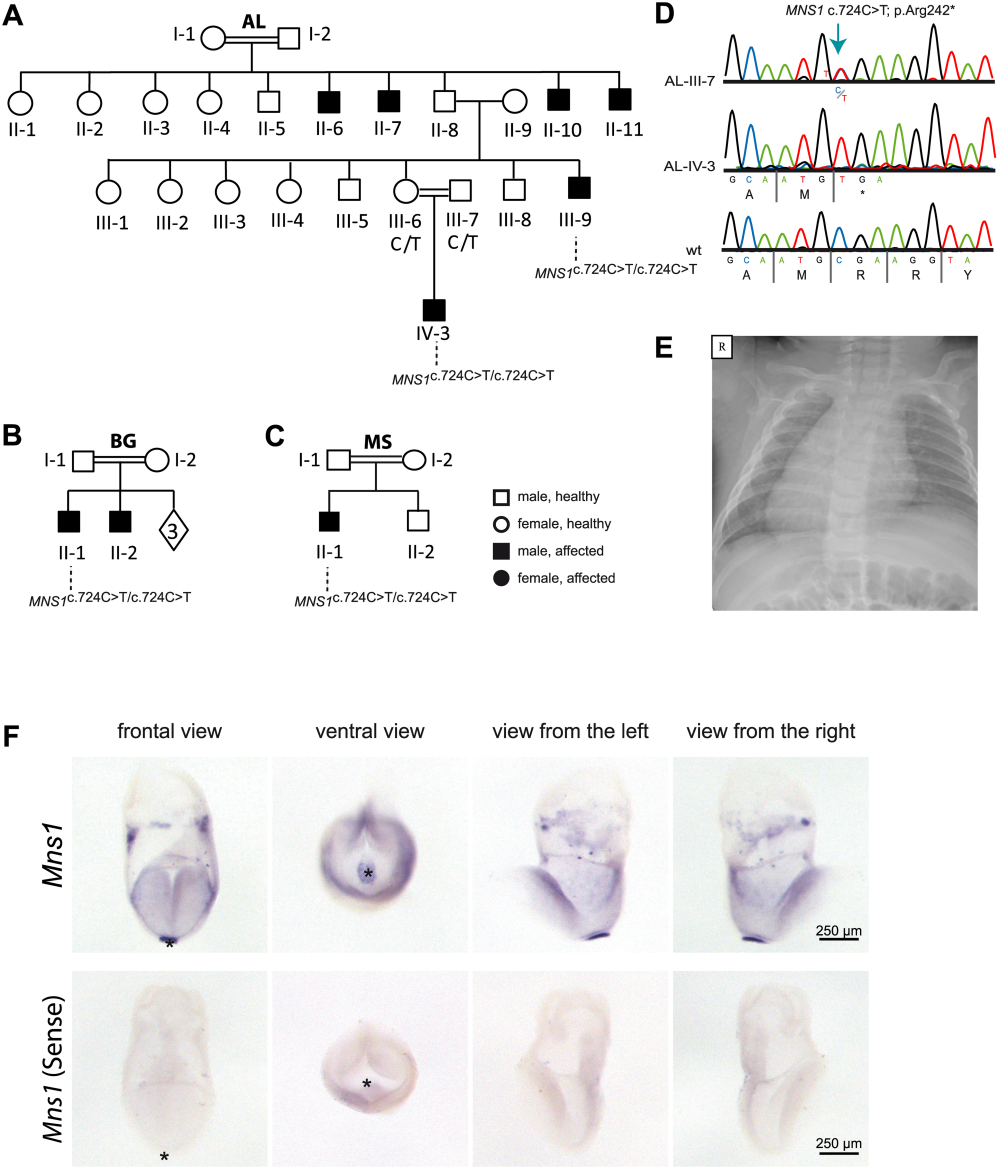
Homozygous loss-of-function mutations in *MNS1* in three consanguineous families with laterality defects. (A-C). Pedigrees of family AL with six affected individuals: AL-II-6, AL-II-7, AL-II-10, AL-II-11, AL-III-9 and AL-IV-3; family BG with two affected individuals: BG-II-1, BG-II-2 and family MS with one affected individual: MS-II-1. In total, 4 males (AL-III-9, AL-IV-3, BG-II-1 ad MS-II-1) carry homozygous loss-of-function *MNS1* mutations (c.724G>A; p.Arg242*). The other affected individuals were not subject to DNA mutation analysis. The parents AL-III-6 and AL-III-7 of the affected individual AL-IV-3, the parents BG-I-1 and BG-I-2 of the affected individual BG-II-1 and the parents MS-I-1 and MS-I-2 of the affected individual MS-II-1 are both carriers of the mutations in a heterozygous state, confirming homozygosity by descent. (D) Bi-allelic *MNS1* nonsense mutations (c.724T>C; p.Arg242*) in a carrier (AL-III-7), an affected individual (AL-IV-3), and a healthy control. (E) Chest X-ray of individual MS-II-1 showing *situs inversus totalis*. R.right. (F) *In situ* hybridization of wildtype mouse embryos detecting *Mns1* expression at the ventral node. In wildtype mouse embryos at 8.25 dpc *Mns1* is expressed predominantly at the ventral node as indicated by the strong blue signal (upper panel). Negative control experiments using the sense probe do not show any specific signal (lower panel). Asterisks in frontal and ventral view mark the position of the ventral node.

Given the genetic heterogeneity of laterality disorders and known genes only explaining a subgroup of cases, we performed independent whole exome sequencing (WES) in 85 affected individuals diagnosed with situs abnormalities with or without heart defects but lacking clinical criteria for PCD, under the hypothesis of a recessively inherited and rare causal allele (Table 1). 8 unrelated families were recruited and evaluated in Israel. In 5 families, more than one member was affected. We performed WES in 8 affected singletons, one from each family. Another 36 families were recruited in Muenster (Germany), and DNA from 37 affected individuals including 2 brothers was analyzed by WES. The remaining 40 Turkish individuals are descendants from 40 families recruited in Turkey and WES was performed and analyzed in the Netherlands. The analysis revealed the same homozygous stop mutation rs185005213, c.724T>C, p.Arg242* in *MNS1* in three affected individuals: 2 from Israel (AL-IV-III, BG-II-1) and 1 from Turkey (MS-II-1) (Fig 1A–1D, Table 1). Lists of variants left after filtering are provided in S1–S4 Tables. Genotyping of all available family members by Sanger sequencing showed segregation of the allele with the disease phenotype in all families, revealing an additional homozygote for this mutant allele: affected individual AL-III-9.

**Table 1:** Overview of the study cohorts analyzed by whole exome sequencing.

Center of Analysis	Israel	Germany	Netherlands	Total
**Families (consanguineous)**	8 (8)	36 (14)	40 (37)	84 (59)
**individuals**	8	37	40	85
**males/females**	7/1	18/19	23/17	48/37
**Age range**	1m–30y	2y–59y	0y–16y	0y-59y
**Origin**	Israel	Germany (27) Israel (8) Switzerland (1) Turkey (1)	Turkey	-
**Inclusion criteria**	Laterality defects, no PCD	Laterality defects, no PCD	Laterality defects, no PCD	-
**Mutations in *MNS1***	c.724T>C; p.Arg242* (2/8)	none	c.724T>C; p.Arg242* (1/40)	3/85

y, years; m, months; PCD: Primary ciliary dyskinesia;

rs185005213 is carried in the heterozygous state by 67 of the ~140,000 healthy individuals whose exome and genome analyses were deposited at gnomAD website (no homozygous LOF individuals were present in this cohort [18]) and has a frequency below 0.1 percent in dbSNP, ExAc, 1000 Genomes Project and the National Heart, Lung and Blood Institute–Exome Sequencing Project.

AL-IV-3 had dextrocardia associated with congenitally corrected transposition of the great arteries, mitral atresia and pulmonic atresia (Table 2). AL-III-9, BG-II-1 and MS-II-1 (Fig 1E) had *situs inversus totalis* without a heart defect and no history of respiratory symptoms (Table 2). Additionally, AL-III-9 and BG-II-1 suffered from infertility. In AL-III-9, the sperm count was reported at 22 X 10^6^ per ml (normal > 15 X 10^6^ per ml). However, under light microscopic examination, less than 1% of sperm cells were found to have normal morphology with the majority having abnormally short tails and some with abnormal head morphology. Furthermore, only 7% of spermatozoa had progressive motility (normal >32%), while 88% did not present any flagellar motility (S5 Table). In BG-II-1, the sperm count was reported at 80 X 10^6^ per ml; only 10% of sperm cells were found to have normal morphology by light microscopy while only 20% of spermatozoa had progressive motility (S5 Table). AL-IV-3 and MS-II-1 are children and precluded sperm analysis.

**Table 2:** Clinical features of the affected individuals.

ID	Homozygous mutations	Sex	Age	Origin	Situs inversus	Infertility	Respiratory symptoms	TEM
AL-II-6	ND	M	55 y	Palestine	ND	Yes	-	ND
AL-II-7	ND	M	53 y	Palestine	ND	Yes	-	ND
AL-II-10	ND	M	47 y	Palestine	ND	Yes	-	ND
AL-II-11	ND	M	44 y	Palestine	ND	Yes	-	ND
AL-III-9	***MNS1***: c.724T>C; p.Arg242*	M	27 y	Palestine	situs inversus totalis	Yes	mild	Partial ODA defect
AL-IV-3	***MNS1***: c.724T>C; p.Arg242*	M	3 m	Palestine	Dextrocardia^a^	ND	-	ND
BG-II-1	***MNS1***: c.724T>C; p.Arg242*	M	29 y	Jordan	situs inversus totalis	suspected	-	ND
BG-II-2	ND	M	26 y	Jordan	situs inversus totalis	Yes	-	ND
MS-II-1	***MNS1***: c.724T>C; p.Arg242*	M	6 y	Turkey	situs inversus totalis	ND	-	ND
OI-11 II1	***DNAH5***:c.13432_13435delCACT; p.His4478Alafs3*	F	ND	Israel	situs inversus totalis	ND	PCD symptoms^b^	ODA defect
OI-11 II6	***MNS1***: c.607C>T; p.Gln203* DNAH5:c.13432_13435delCACT; p.His4478Alafs3*	F	15 y	Israel	situs inversus totalis	ND	PCD symptoms^b^	ODA and ODA-DC defect
OI-14 II1	***DNAH5***:c.13432_13435delCACT; p.His4478Alafs3*	F	24 y	Israel	Situs solitus	ND	PCD symptoms^b^	ODA defect
OI-24 II1	***DNAH5***:c.13432_13435delCACT; p.His4478Alafs3*	M	17 y	Israel	situs inversus totalis	ND	PCD symptoms^b^	ODA defect
OI-24 II2	***DNAH5***:c.13432_13435delCACT; p.His4478Alafs3*	F	16 y	Israel	situs inversus totalis	ND	PCD symptoms^b^	ODA defect

M: Male; F: Female; y, years; m, months; TEM: Transmission electron microscopy; PCD: Primary ciliary dyskinesia; ODA: Outer dynein arm; ODA-DC: Outer dynein arm docking complex; ND: Not determined;

-: absence.

^a^associated with congenitally corrected transposition of the great arteries, mitral atresia and pulmonic atresia.

^b^PCD symptoms: bronchiectasis, recurrent pneumonia, recurrent sinusitis, recurrent otitis media and chronic cough.

Thus, homozygous *MNS1* mutations in humans result in laterality defects and likely cause infertility, consistent with the phenotype of *Mns1*^*-/-*^ mouse [17].

### *Mns1* is expressed at the mouse embryonic node

In humans and mice, *MNS1*/*Mns1* mutations are associated with laterality defects; therefore, we hypothesized that MNS1 may be essential for the embryonic nodal function as left/right organizer during early embryonic development, either playing a role for nodal monocilia function or for cilia independent processes in the embryo. To verify nodal expression, we performed *in situ* hybridization analyses of 8,25 dpc (days post coitum) mouse embryos (Fig 1F). At this stage, the left/right organizer or ventral node is present at the posterior end of the midline and the left/right body axis develops. By utilizing a probe complementary to *Mns1* mRNA, we detected expression of *Mns1* within the gastrulating mouse embryo. Compared to other embryonic tissues at this developmental stage, *Mns1* expression is strongly enriched in the ventral node (Fig 1F).

### MNS1 localizes to human respiratory ciliary axonemes and sperm flagella

**Fig 2:**
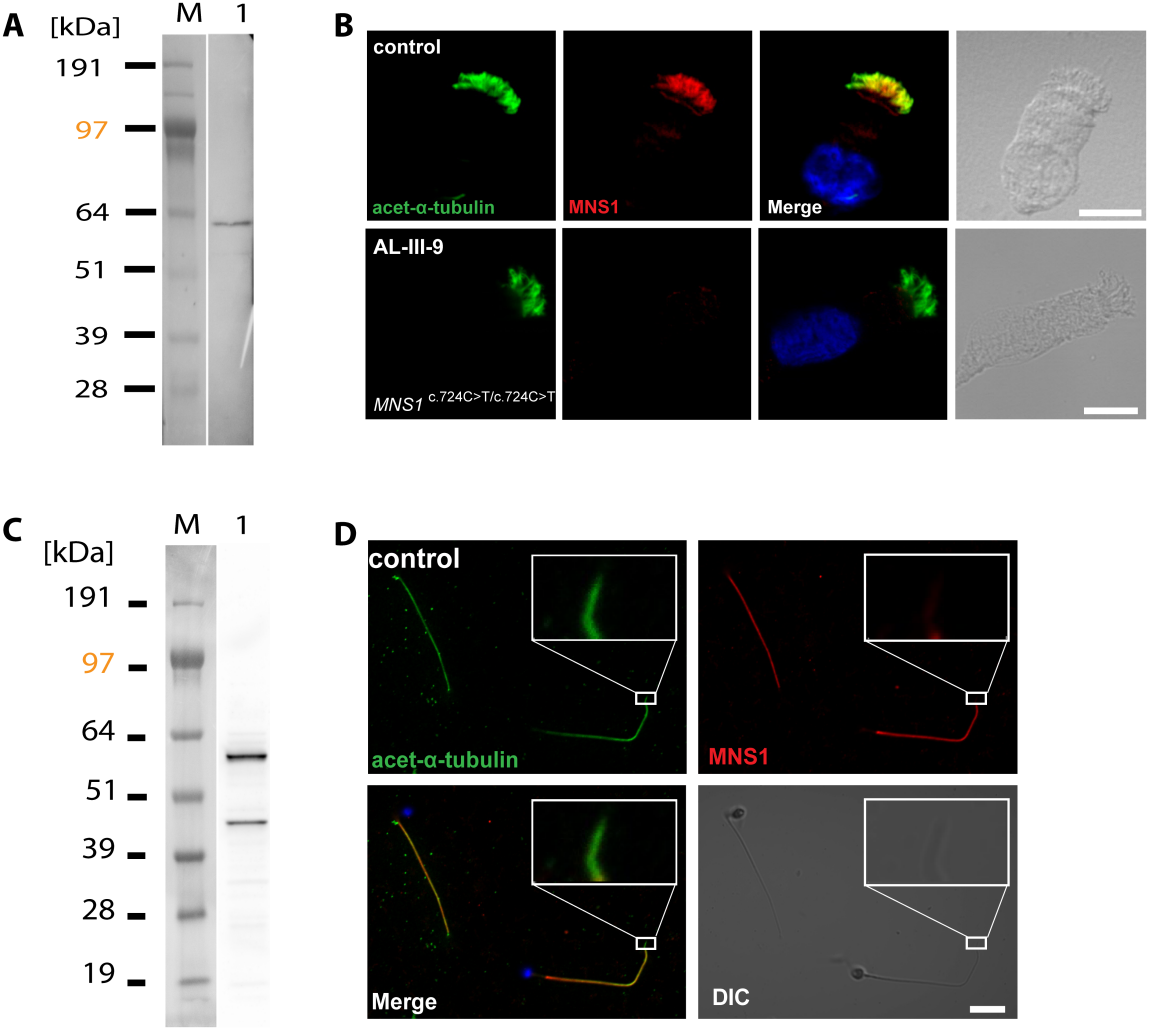
MNS1 localizes to human respiratory cilia and human sperm flagella. (A) Western blot analysis of protein lysates from human respiratory cells (M, protein standard). MNS1 antibodies specifically detect a single band with the predicted size (~61kDa, lane 1). (B) Respiratory epithelial cells from control and affected individual AL-III-9 carrying bi-allelic *MNS1* mutations. Cells were double-labeled with antibodies directed against acetylated alpha-tubulin (green) and MNS1 (red). Nuclei were stained with Hoechst 33342 (blue). Both proteins co-localize (yellow) along the ciliary axonemes in cells from the unaffected controls, while in respiratory cells of AL-III-9, MNS1 is undetectable in the ciliary axonemes, consistent with recessive loss-of-function *MNS1* nonsense mutations. Scale bars, 10μm. (C) Western blot analysis of lysate from human whole sperm cells (M, protein standard). Anti-MNS1 antibody specifically detects a band at the predicted size (~61kDa, lane 1) and a band of approximately ~45kDa, indicating an isoform of MNS1 in sperm. (D) In human control spermatozoa, MNS1 (red) co-localizes with acetylated alpha-tubulin (green) along flagellar axonemes except at the endpiece (white box).

Because we detected *Mns1* expression in the ventral node of mouse embryos, indicating a potential role of MNS1 for node monocilia function, we went further to check if MNS1 is expressed in human ciliary tissues as the axonemes of respiratory epithelial cells and sperm flagella. Using a rabbit polyclonal antibody specific to MNS1, we found by Western blot analysis that MNS1 is expressed in human nasal respiratory epithelial cells and in whole sperm lysates (Fig 2A and 2C). This antibody recognized a single protein with the expected size (60 kDa) in both lysates, while one additional band of approximately 45 kDa was detected in sperm lysates (Fig 2C), likely indicating an isoform of MNS1. This isoform likely corresponds to the sequence listed in AceView, cloned from testis with 363 amino acids and with a molecular weight of 45 kDa (AK057542).

Moreover, we analyzed the localization of MNS1 in human motile respiratory cilia by immunofluorescence microscopy (IF) and determined that in contrast to the control (Fig 2B), MNS1 was undetectable in the respiratory cilia of individual AL-III-9, thus confirming the LOF mutations in *MNS1* and supporting antibody specificity. We subsequently determined by IF that MNS1 is detectable in spermatozoa flagella from human control sperm, prominently localizing to the midpiece and the principal piece (Fig 2D).

### Identification of *MNS1* mutations through whole genome linkage and haplotype analysis

**Fig 3:**
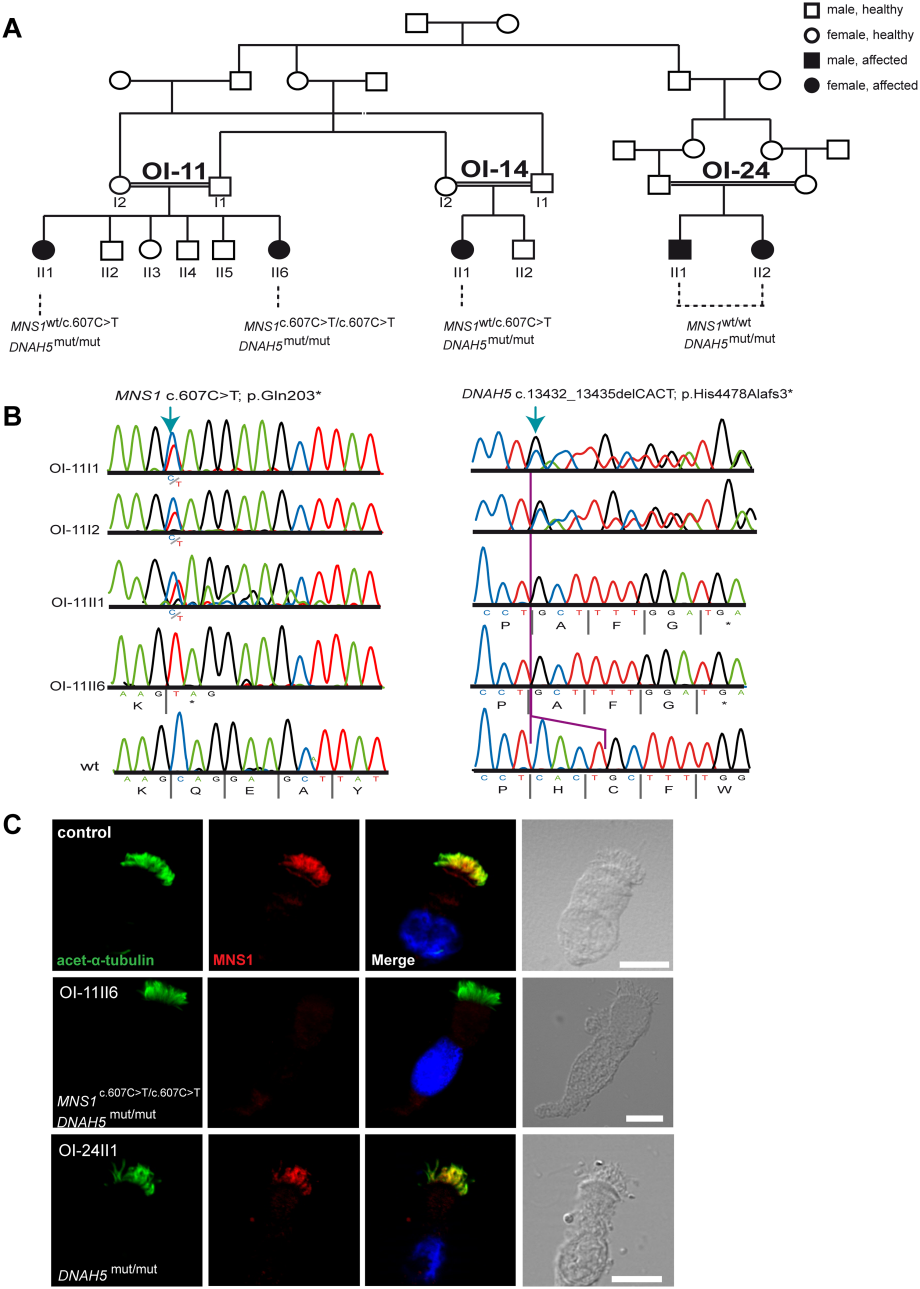
Identification of *MNS1* loss-of-function mutations in a PCD-affected individual with *DNAH5* mutations. (A) Pedigree of families OI-11, OI-14 and OI-24: consanguinity of first degree. In total, five PCD-affected individuals carry homozygous mutations in *DNAH5* (annotated as *DNAH5*^*mut/mut*^) of whom only one (OI-11 II6) carries additional homozygous mutations in *MNS1* (c.607C>T; p.Gln203*). (B) Bi-allelic *MNS1* nonsense mutations in OI-11 II6 (c.607C>T) predicting a premature termination of translation (p.Gln203*). The affected sibling OI-11 II1 and both parents OI-11 I1 and I2 are carriers of the mutant allele. *Right panel*. Bi-allelic *DNAH5* nonsense mutations in OI-11 II6 and OI-11 II1 (c. c.13432_13435delCACT) predicting a premature termination of translation (p.His4478Alafs3*). Both parents OI-11 I1 and I2 are carriers of the mutant allele. (C) Respiratory epithelial cells from control and affected individuals: OI-11 II6 carrying bi-allelic *MNS1* and *DNAH5* mutations, and OI-24 II1 carrying the identical bi-allelic *DNAH5* mutations as OI-11 II6. For space issues, OI-24 II1 is referred to in this and other Figures as *DNAH5*^*mut/mut*^ instead of *DNAH5*^*c*.*13432_13435delCACT/ c*.*13432_13435delCACT*^. Cells were double-labeled with antibodies directed against acetylated alpha-tubulin (green) and MNS1 (red). Nuclei were stained with Hoechst 33342 (blue). Both proteins co-localize (yellow) along the ciliary axonemes in cells from the unaffected controls and OI-24 II1, while in respiratory cells of OI-11 II6, MNS1 is undetectable in the ciliary axonemes, consistent with recessive loss-of-function *MNS1* nonsense mutations. Scale bars, 10μm.

Moreover, we analyzed whole genome linkage and haplotype analysis data in our PCD cohort recruited in Muenster (Germany) on the basis of respiratory symptoms, laterality defects and/or infertility. Linkage analysis was performed in 161 different consanguineous families. In only 4 families, homozygosity maps revealed homozygous regions encompassing *MNS1*. After Sanger sequencing of all exons in 4 affected individuals (one from each family), we identified in individual OI-11 II6 a homozygous nonsense mutation (c.607C>T) in *MNS1* predicting a premature termination of translation (p.Gln203*). OI-11 II6 is descendant of first-degree consanguineous parents from the multiplex Israeli family (Fig 3A and 3B, S1 Fig). Both unaffected parents were heterozygous for this mutation (Fig 3B). The detected mutation in *MNS1* is not listed in gnomAD, absent in 180 controls of European ancestry and not found in the dbSNP, ExAc, 1000 Genomes Project or the National Heart, Lung and Blood Institute–Exome Sequencing Project human polymorphism databases.

X-ray analysis showed that OI-11II6 has *situs inversus totalis*, consistent with findings reported in *Mns1*^*-/-*^ mice, in which the genetic defect causes randomization of left/right body asymmetry [17]. This individual is a 15 year old female and could not be assessed for fertility. OI-11 II1, the other affected sibling, was heterozygous for this mutation.

Whereas the affected individuals AL-IV-3, AL-III-9, BG-II-1 and MS-II-1 (described above) did not present a respiratory phenotype, OI-11 II6 exhibited classical PCD symptoms (Table 2), indicating that she might be carrying additional mutations in a PCD-related gene.

In PCD, the most frequent abnormalities affect ODA composition and function. Mutations in the axonemal heavy chain dynein gene *DNAH5* (MIM 603335) cause PCD with ODA defects and account for more than 50% of the total ODA deficiency cases [19–20]. In effect, by screening the five hot-spot exons of *DNAH5* [21], we also identified a homozygous deletion of four nucleotides in *DNAH5* (c.13432_13435delCACT) leading to a frameshift and premature termination of translation (p.His4478Alafs3*) in OI-11 II6 (Fig 3A and 3B). Both parents were heterozygous carriers for this *DNAH5* mutation (Fig 3B). Thus, OI-11 II6 carries bi-allelic homozygous LOF mutations in both *MNS1* and *DNAH5*. Moreover, we identified the identical homozygous frameshift *DNAH5* mutations in OI-11 II1 (sister of OI-11 II6), OI-14 II1, OI-24 II1 and OI-24 II2 (PCD-affected individuals from two consanguineous families closely related to family OI-11, Fig 3A).

We then analyzed MNS1 localization in respiratory cells of OI-11 II6 (mutations in *DNAH5* and *MNS1*) and OI-24 II1, which carries the same *DNAH5* mutations as OI-11 II6 but is wildtype for *MNS1* (Fig 3C). As expected, MNS1 was absent from OI-11 II6 axonemes. Interestingly, MNS1 localization in *DNAH5* mutant cells (OI-24 II1) was normal, indicating that the ODA protein DNAH5 is not essential for axonemal MNS1 localization.

Because ODA defects were reported in *Mns1*-deficient mice, we subsequently sequenced all exons of *MNS1* in 134 PCD-affected individuals from different origins (60 from Germany, 45 from Israel, 24 from Denmark, 2 from Hungary, 2 from Greece and 1 from Turkey). These individuals were characterized to have an ODA defect by TEM and/or by IF and with no mutations in reported PCD-related genes. Interestingly, no *MNS1* mutations could be found in any of these individuals.

### Isolated MNS1 deficiency results in subtle ODA defects while a combined MNS1 and DNAH5 deficiency results in ODA-DC defect

**Fig 4:**
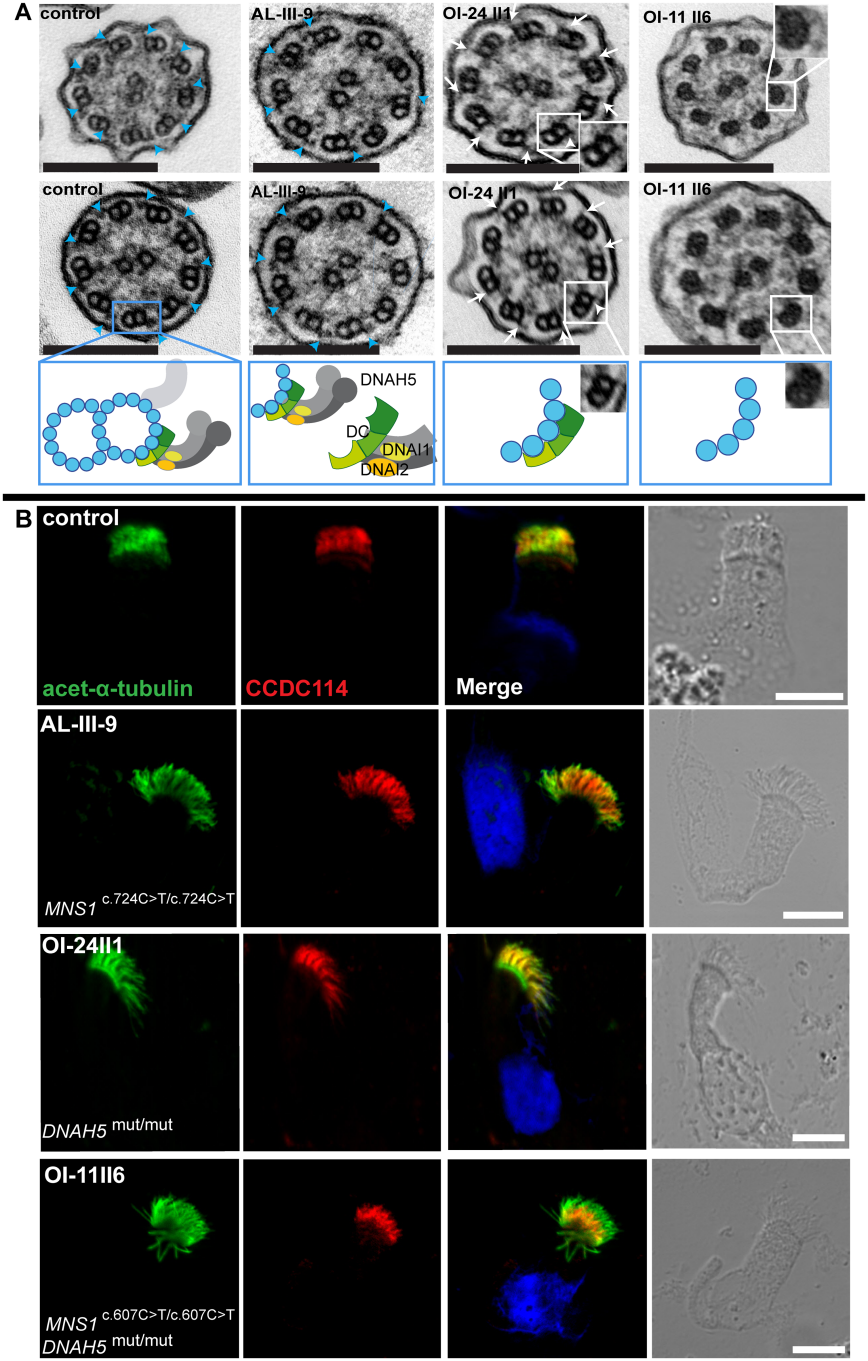
Mutations in *MNS1* when combined with mutations in *DNAH5* might result in defects of the ODA-microtubule docking complex in human respiratory epithelial cells. (A) Transmission electron micrographs show subtle ultrastructural defects in affected individual AL-III-9 carrying bi-allelic *MNS1* mutations with the occasional absence of only few ODAs (2–4 out of 9) in about half of the cross-sections (compared to control samples where all analyzed sections show an average of 8.7 ODAs, 9 analyzed sections from the MNS1-deficient ciliary axonemes show an average of 6 ODAs). However, TEM show complete absence of ODAs in PCD-affected individuals OI-24 II1 (*DNAH5* mutations) and OI-11 II6 (*MNS1* and *DNAH5* mutations) compared to a control without PCD. In the healthy control, outer dynein arms are visible (blue arrows). However, the cilia from OI-24 II1 still have the ODA-DC (small projections marked by white arrows) whereas the cilia from OI-11 II6 do not, suggesting that MNS1 deficiency when combined with DNAH5 deficiency might cause defects in ODA-DC assembly. Below the control TEM section a schematic illustrating a microtubular doublet with attached ODA docking complex (ODA-DC) and the double-headed ODA complex proteins with dynein heavy chain DNAH5 and dynein intermediate chains DNAI1 and DNAI2. In affected individual AL-III-9, a partial defect is observed; in OI-24II1, a schematic where the ODA complex is absent while the ODA-DC is still retained; in OI-11II6, a schematic where both ODA and ODA docking complexes are absent. Scale bars, 0.1 μm. (B) Respiratory epithelial cells from control and affected individuals: AL-III-9 carrying bi-allelic *MNS1* mutations, OI-11 II6 carrying bi-allelic *MNS1* and *DNAH5* mutations and OI-24 II1 carrying no mutations in *MNS1* but identical bi-allelic *DNAH5* mutations as OI-11 II6. For space issues, OI-24 II1 is described as *DNAH5*^*mut/mut*^ instead of *DNAH5*^*c*.*13432_13435delCACT/ c*.*13432_13435delCACT*^. Cells were double-labeled with antibodies directed against acetylated alpha-tubulin (green) and CCDC114 (HPA042524, Atlas antibodies) (red). Nuclei were stained with Hoechst 33342 (blue). Both proteins co-localize (yellow) along the ciliary axonemes in cells from the unaffected controls, AL-III-9 and OI-24 II1, while in cells of OI-11 II6, CCDC114 localizes only to the proximal part of the ciliary axonemes, indicating that recessive loss-of-function mutations in *MNS1* when combined with loss-of-function mutations in *DNAH5* might affect the distal localization of ODA-DC associated proteins and might play a role in docking or anchoring the ODA subunits or in regulating this process. Scale bars, 10μm.

Furthermore, we analyzed respiratory cilia of individual AL-III-9 (*MNS1* mutations) and OI-11 II6 (combined *MNS1* and *DNAH5* mutations) by TEM. The ciliary ultrastructure of individual AL-III-9 displayed a slight reduction of ODAs attached to outer doublets (up to 3–4 ODAs missing out of 9), indicating a partial defect of ODA assembly (control mean ODA: 8.7; affected individual mean ODA: 6). As expected, TEM of individuals OI-24 II1 (*DNAH5* mutations) and OI-11 II6 (combined *MNS1* and *DNAH5* mutations) showed absent ODAs (Fig 4A), a typical finding caused by *DNAH5* mutations [19].

Further IF analyses with antibodies targeting DNAH5 [19] and the axonemal ODA intermediate chains DNAI1 [21] and DNAI2 [22] confirmed normal axonemal localization of the three ODA proteins in respiratory cilia of individual AL-III-9 (*MNS1* mutations), whereas in respiratory cells of individual OI-11 II6 (combined *MNS1* and *DNAH5* mutations), DNAH5 as well as DNAI1 and DNAI2 were completely absent from ciliary axonemes (S2 and S3 Figs), as expected due to the *DNAH5* mutations and consistent with the ODA defects as demonstrated by TEM. Normal axonemal localization of the inner dynein arm (IDA) light chain DNALI1 and the nexin dynein regulatory complex (N-DRC) component GAS8 (S3 Fig) confirmed absence of other ciliary ultrastructure defects due to *MNS1* mutations. Accordingly, PCD-affected individual OI-24 II1 (biallelic *DNAH5* mutations without *MNS1* impairment) showed the same pattern of staining as OI-11 II6 (biallelic *DNAH5* and *MNS1* LOF mutations) (S2 and S3 Figs). These findings are consistent with reported PCD-affected individuals with *DNAH5* mutations [19–20].

Interestingly, by carefully analyzing ultrastructural cross-sections of OI-24 II1 (biallelic *DNAH5* mutations without *MNS1* impairment) by TEM, we noticed residual projections on the doublet microtubules (Fig 4A, white arrows), known as the projections of the ODA docking complex (ODA-DC) system, which facilitate the attachment of ODAs onto microtubules [23] but are not affected by *DNAH5* mutations. The same residual ODA-DC projections were present in *DNAH5*-mutant cilia from other PCD individuals with bi-allelic *DNAH5* mutations [24]. In contrast, TEM analysis of OI-11 II6 (*DNAH5* and *MNS1* mutations) showed these projections were absent, demonstrating that a combined *DNAH5* and *MNS1* deficiency likely disrupts correct ODA-DC assembly.

### *MNS1* functionally associates with ODAs via its interaction with the ODA docking complex (ODA-DC) component CCDC114

To address whether MNS1 supports ODA-DC function, we examined the localization of the ODA-DC component CCDC114 [25–26] in respiratory cilia of individuals AL-III-9 (*MNS1* mutations), OI-24 II1 (*DNAH5* mutations) and OI-11 II6 (combined *MNS1* and *DNAH5* mutations). In control as well as in MNS1-deficient cilia of individual AL-III-9 and DNAH5-deficient cilia of OI-24 II1, CCDC114 normally localizes along the entire axonemes (Fig 4B). In contrast, CCDC114 staining is reduced in combined *MNS1-* and *DNAH5-*mutant cilia, where it localized only to the proximal region of axonemes (Fig 4B). In addition, we examined the localization of the ODA-DC associated protein ARMC4 [27] in respiratory cilia of all three individuals mentioned above. ARMC4 localized normally in MNS1-deficient cilia and in DNAH5-deficient cilia but like CCDC114, it was absent in the distal axonemes of cilia with both *MNS1* and *DNAH5* mutations (S4 Fig). Thus, the combined deficiency of MNS1 and DNAH5 results in abnormal ODA-DC assembly (proximal CCDC114 and ARMC4), consistent with the absence of ODA-DC projections in individual OI-11 II6 detected by TEM.

**Fig 5:**
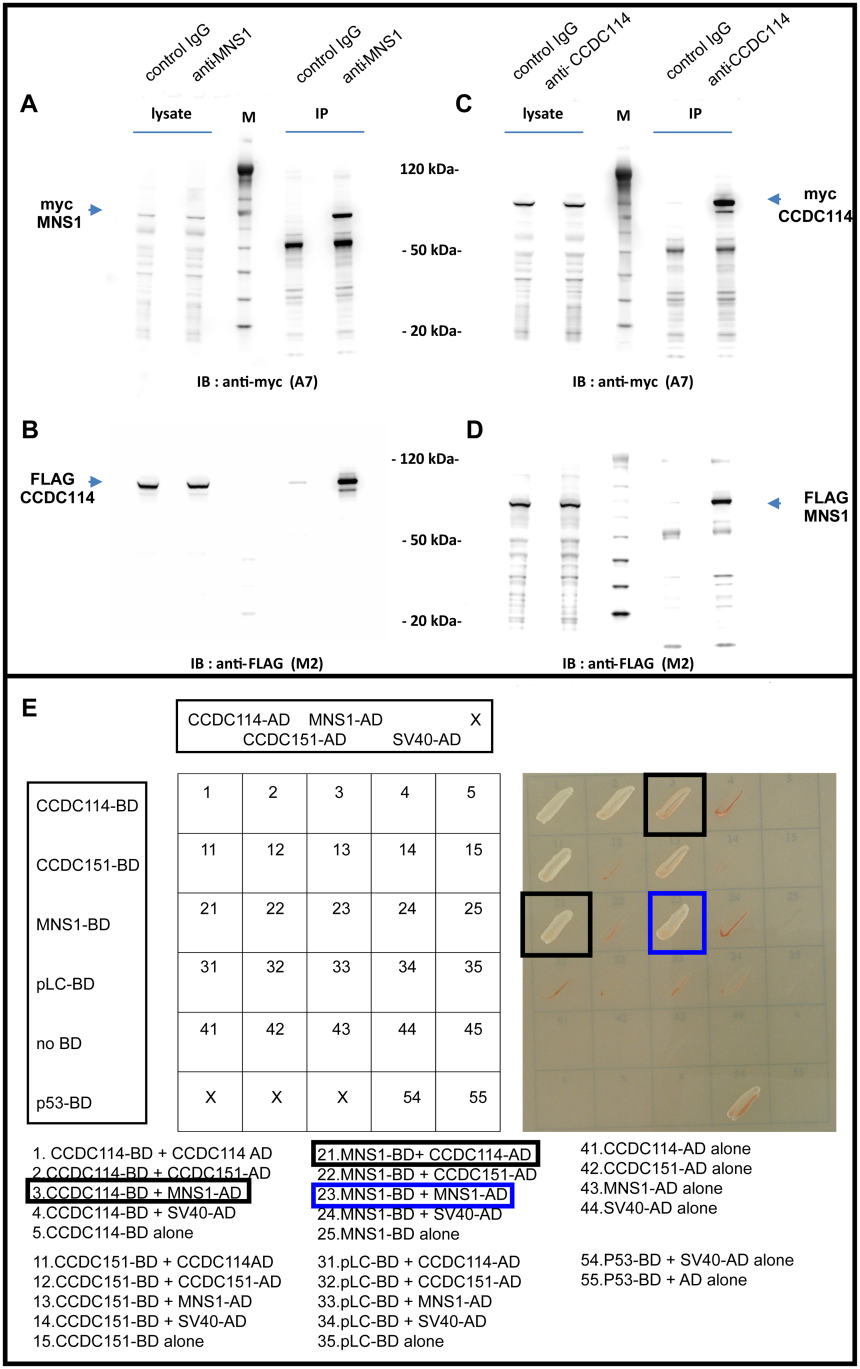
MNS1 forms a dimer and interacts directly with the ODA-DC protein CCDC114. HEK293 lysates coexpressing FLAG or myc epitope-tagged MNS1 and CCDC114 were immunoprecipitated with either rabbit control IgG and rabbit anti-MNS1 or anti-CCDC114 antibody. Western blotting with mouse anti-myc or anti-FLAG antibody demonstrates that MNS1 immunoprecipitates CCDC114 (A-B) and CCDC114 immunoprecipitates MNS1 (C-D). The open reading frames of recombinant MNS1 and CCDC114 are 495 and 670 amino acids, respectively. The observed approximate molecular weights of 70 and 85 kDa, respectively, represent additional sequence from myc- and FLAG epitope tags. Equal volumes (12 μl) of lysate and immunoprecipitate fractions were loaded on the same gel; lysate fractions represent 0.7% of total lysate (1 ml volume) and immunoprecipitate fractions represent 1/15 lysis volume (33 μl resuspension). Magic Mark protein ladder (M) was used to estimate molecular weight of recombinant MNS1 and CCDC114. (E) Yeast two-hybrid screening of MNS1 and CCDC114 reveals direct protein interaction between MNS1 and CCDC114 (black squares) and dimerization of MNS1 (blue square). Interactions were analyzed by assessment of reporter gene (*HIS3* and *ADE2*) activation via growth on selective media (SD-LWAH) and β-galactosidase colorimetric filter lift assays (*LacZ* reporter gene). A dimerization of CCDC114 (block 1) and a direct protein interaction between CCDC114 and CCDC151 (blocks 2 and 11) as demonstrated previously by Hjeij et al. 2014, are also here demonstrated.

Considering that the ODA-DC protein CCDC114 assembly was absent from the distal ciliary axonemes in respiratory cells with a combined MNS1 and DNAH5 deficiency, we tested for possible interactions between MNS1 and ODA and ODA-DC proteins including CCDC114. Using myc- and FLAG-tagged proteins that were co-expressed in HEK293 cells, we found by co-immunoprecipitation that MNS1 interacted with CCDC114 (Fig 5A–5D) but not with ODA proteins DNAI1, DNAI2, and DNAL1, that when mutated also result in ODA defects. We confirmed the reciprocal interaction between CCDC114 and MNS1 by yeast two-hybrid analysis (Y2H) (Fig 5E). We could also show by Y2H that human MNS1 self-dimerized, in agreement with the report of mouse MNS1 dimerization [17] (Fig 5E). Overall, these data indicate that MNS1 plays also a functional role in ODA docking.

## Discussion

Consanguineous families are a resource for the identification of disease-associated genes. Within the framework of our cardio-genetic project, we have identified several CHD genes [28–29] and reached a diagnosis in ~70% of the study families. Here we report the identification of LOF mutations in *MNS1* in individuals with laterality defects ranging from *situs inversus totalis* to dextrocardia with congenitally corrected transposition of the great arteries and mitral atresia (see Table 2). Zhou *et al*. reported laterality defects in *Mns1*-deficient mice: accordingly, of 36 *Mns1*^*-/-*^ mice tested for laterality defects, 8 (22%) presented with *situs inversus totalis*, 6 (17%) exhibited left isomerism and the remaining had *situs solitus* [17]. Because approximately half of the mutant mouse offspring (39%) exhibited laterality defects at birth, we assume that the genetic defect causes randomization of left/right body asymmetry, as observed in other mouse models with abnormal nodal ciliary motility function [27;30]. Our observation of *Mns1* expression at the mouse left/right organizer and that mutations in *MNS1*/*Mns1* in human and mouse are associated with laterality defects indicates that MNS1 might be a functional component of motile node monocilia relevant for laterality development.

MNS1 was reported by western blot to be highly expressed in mouse testis, consistent with the encoded protein being a component of the sperm flagella [31]. Using WB and IF analyses, we detected two isoforms of MNS1 and determined the localization of MNS1 throughout the human sperm flagellum, predominantly in the mid and principal piece where the accessory structures are located. Male *Mns1*-deficient mice were sterile, and their epididymis sperm count was reduced to only 8% of the wild type level [17]. Most of the sperm cells had very short tails with no motility while the head morphology appeared normal [17]. Two affected males in this study have infertility problems with severely reduced flagellar motility and abnormal morphology of the sperm tails. The third and fourth males are still too young for analysis. Interestingly, five males related to the reported individuals with *MNS1* mutations (AL-III-9 and BG-II-1) suffered also from infertility, but these individuals were not included in our genetic analysis.

Respiratory cilia in humans with *MNS1* mutations displayed only partial ODA defects, similar to previously reported findings in *Mns1*-deficient mice [17]. Most ODAs were still present in patient respiratory cilia, indicating that their ciliary motility was not significantly altered, consistent with only mild or no respiratory complaints reported in MNS1-deficient individuals.

We have previously shown that while ODA-DC deficiency affects the attachment of ODAs to the axonemes, deficiency of ODAs does not affect ODA-DC assembly [24; 27; 30]. Here we observed for the first time that ODA (DNAH5) deficiency causes a defect in ODA-DC assembly if accompanied with MNS1 deficiency. Analyses in a PCD-affected individual with combined MNS1 and DNAH5 deficiency showed that the ODA-DC protein CCDC114 and the ODA-DC associated protein ARMC4 were absent from the distal ciliary axonemes, suggesting that MNS1 functions in ODA docking. We have previously reported at least two discernible ODA types in ciliary axonemes of human respiratory cilia -type 1 proximal compartment that contain DNAH5 and DNAH11, and type 2 distal compartments that contain DNAH5 and DNAH9 [32–33]. Our observation that a combined MNS1 and DNAH5 deficiency affects CCDC114 and ARMC4 localization only in the distal axonemes raises the possibility that distinct docking complexes exist for the two types of ODAs in humans.

It was recently shown that the ODA-DC is more likely to be a flexible stabilizer of ODAs where it strengthens the electrostatic interactions between ODAs and microtubules, rather than a molecular ruler on which ODAs attach [34]. In contrast to the prior assumption that ODA-DC is an intermediate between ODAs and microtubules, it seems possible that ODAs at this point also interact directly with microtubules. Thus, ODAs, ODA-DC and microtubules interact altogether to stabilize both the attachment of ODAs and the attachment of ODA-DC to the microtubules. Based on the data presented in this paper, we can hypothesize that in the distal axonemes, a complex involving MNS1, ODA-DC and microtubules might be also playing a role in stabilizing the attachment of ODA-DC to the microtubules. When ODAs (DNAH5) are absent, MNS1 could still maintain the attachment of ODA-DC to the microtubules and when MNS1 is absent, ODAs (DNAH5) could also maintain the attachment of ODA-DC to the microtubules at the distal part of the cilia. However, when both ODAs (DNAH5) and MNS1 are absent, ODA-DC fails to assemble in the distal axonemes, indicating that DNAH5 and MNS1 compensate for each other in the stabilization of ODA-DCs to the microtubules at the distal part of the cilia.

The physical interaction between MNS1 and CCDC114 demonstrated here by IP and Y2H supports this hypothesis. Additional intermediate partners interacting with MNS1, CCDC114 and DNAH5 that contribute to correct axonemal localization of ODAs and the regulation of ODA-docking in mammals remain to be determined.

In summary, we identify *MNS1* mutations as the cause of laterality defects as well as reduced male fertility and identify an axonemal and flagellar localization of MNS1 in human respiratory cilia as well as sperm cells. We confirm partial outer dynein arm (ODA) abnormalities in respiratory cilia from an MNS1-deficient individual, as observed in *Mns1*^*-/-*^ mice. Analyses indicate that MNS1 likely functions also in the process of outer dynein arm docking (ODA-DC). Further functional analyses regarding MNS1 function will help to better understand the molecular mechanisms involved in laterality defects as well as male infertility.

## Materials and methods

### Ethics statement

All research complies with the ethical standards in Israel. The ethical committees of Hadassah Medical Center and the Israeli Ministry of Health approved this study; approval number is 0306-10-HMO. All research complies with the ethical standards in Germany and with the rules as set by the European Union; the local Ethics committee Ethikkommision der Ärztekammer Westfalen-Lippe und der Medizinischen Fakultät der Westfälischen Wilhelms-Universität approved this study, approval number is 2010-298-b-S. WES in Nijmegen was perfomred diagnostically in the Human genetics department under the Innovative Diagnostics programme.

Prior to participation, written informed consent was obtained from all patients and family members.

All experiments utilizing animals were performed under the approval of local government authorities (Landesamt für Natur, Umwelt und Verbraucherschutz Nordrhein-Westfalen, Germany (Az.: 84–02.05.20.12.164, and Az.: 84–02.05. 5.15. 012).

### Subjects

This study involved 85 individuals with laterality defects without evidence of PCD. Nine affected individuals -whose clinical data are presented in Table 2- originating from three unrelated consanguineous families, are presented here (Fig 1A–1C):

- Family AL is a four-generation Palestinian family (Fig 1A) including six affected individuals:
  Affected individual AL-IV-3 is a male born at term with a low birth weight of 2.3 Kg. Severe CHD was suspected during gestation. At birth he presented with central cyanosis and heart murmur but no other pathological findings on physical examination. Echocardiography demonstrated dextrocardia, congenitally corrected transposition of the great arteries, normal systemic and pulmonary venous return, atrio-ventricular discordance, mitral atresia, ventriculo-arterial discordance and pulmonic atresia. At the age of 5 days, he underwent Blalock–Taussig shunt (BTS), ligation of persistent ductus arteriosus and atrial septectomy. When last seen, at 3 months of age, he had mild failure to thrive (FTT).
  Affected individual AL-III-9, the maternal uncle of AL-IV-3, was diagnosed incidentally at the age of 15 years with *situs inversus totalis* after a chest x-ray was indicated due to mild respiratory illness. There was no past medical history of chronic cough, sinusitis, rhinitis or recurrent ear infections. His cardiac anatomy and function were normal. Nasal nitric oxide measurement was normal (1,231 nl / min) as was his lung clearance index (6.75, predicted—6.69) measured by multiple N_2_ breath washouts. He suffers additionally from infertility.
  Affected individuals AL-II-6, AL-II-7, AL-II-10, and AL-II-11 were examined only after the molecular diagnosis of the index individual; all were married adults who suffered from infertility, necessitating multiple IVF cycles. Their medical history was negative for respiratory, nasal or ear diseases as well as for congenital cardiac defects; their situs condition was not assessed.
- Family BG is a consanguineous family from Jordan with 2 affected individuals:
  Individuals BG-II-1 (Fig 1B) and BG-II-2 had *situs inversus totalis* without structural heart defect and suffer from infertility. While B-II-1 did not show significant respiratory symptoms, BG-II-2 had nasal polyps and episodes of sinusitis.
- Family MS is a consanguineous Turkish family with one affected individual:
  MS-II-1 (Fig 1C) is 6 years old, had *situs inversus totalis* without structural heart defect or significant respiratory symptoms.

On the basis of the recurrence of similar clinical features, and reportedly healthy parents, we hypothesized an autosomal-recessive mode of inheritance in the families mentioned. To investigate the molecular underpinnings of disease, we collected DNA samples after obtaining informed patient or parental consent. The study was performed with the approval of the ethical committees of Hadassah Medical Center and the Israeli Ministry of Health as well Turkish ethics committee and Institutional Ethics Review Board at the University of Muenster.

In parallel, we searched our large PCD cohort in Muenster (Germany) for consanguineous families with linkage analysis showing big homozygous regions on chromosome 15 encompassing *MNS1*. We found 4 consanguineous families (3 from Israel and 1 from Germany) showing linkage to *MNS1* and performed Sanger sequencing in all affected individuals.

Among these families, family OI-11 is a part of a large Israeli consanguineous family with 5 affected individuals (Fig 4 and Table 2):

Individual OI-11 II6 is a 15 year old girl exhibiting *situs inversus totalis* and classical PCD symptoms including bronchiectasis, recurrent pneumonia, recurrent sinusitis, recurrent otitis media and chronic cough. Nasal NO levels were very low (6 nl/min), consistent with PCD [35]. Individual OI-11 II1 shared the same symptoms with her sister OI-11 II6.

OI-14 II1, OI-24 II1 and OI-24 II2 from two Israeli consanguineous families related to family OI-11, had the same respiratory symptoms as OI-11 II6.

Independently, based on phenotypic data including laterality defects, infertility and ODA defects reported in *Mns1*-deficient mice, we considered *MNS1* a reasonable candidate for PCD in humans. Subsequently, we screened 134 PCD-affected individuals characterized to have an ODA defect documented by transmission electron microscopy (TEM) and/or by immunofluorescence analysis (IF) for mutations in *MNS1*.

### Whole exome analysis

Exomic sequences from DNA samples of AL-IV-3 and BG-II-1 were enriched with the SureSelect Human All Exon 50 Mb V.5 Kit (Agilent Technologies, Santa Clara, California, USA) and SureSelect Human All Exon V.6 Kit was used for sample MS-II-1. AL-IV-3 and BG-II-1 sequences (100-bp paired-end) were generated on a HiSeq2500 and MS-II-1 sequences were generated on a Hiseq PE150 (Illumina, San Diego, California, USA). Read alignment and variant calling were performed with DNAnexus (Palo Alto, California, USA) using default parameters with the human genome assembly hg19 (GRCh37) as reference.

The analysis in affected individuals AL-IV-3 and BG-II-1 from consanguineous families A (Palestine) and B (Jordan) yielded 43.0 and 47.3 million mapped reads with a mean coverage of 84x and 82x, respectively. Following alignment and variant calling, we performed a series of filtering steps. In AL-IV-3 and BG-II-1, we excluded variants which were called less than 8x, off-target, heterozygous, synonymous, and had MAF>0.5% at ExAC (Exome Aggregation Consortium, Cambridge, MA) or MAF>2% at the Hadassah in-house database (~1000 ethnic matched exome analyses). In MS-II-1, the following filters are used: MAF 0.5% in ExAc, 1000 genome project and esp6500 databases, coding variant or within 20 bp of exon-intron boundaries, genes carrying bi-allelic variants with prioritization of stop homozygous variants.

### Haplotype analysis and genome-wide SNP mapping

We performed total-genome scans by using single-nucleotide polymorphism (SNP) arrays (Affymetrix GeneChip Human Mapping 10K Array v.2.0 [Affymetrix, Santa Clara, CA, USA]) in 161 consanguineous families. Relationship errors were evaluated with the help of the program Graphical Relationship Representation. The program PedCheck was applied for detecting Mendelian errors. Non-Mendelian errors were identified with the program MERLIN. Linkage analysis was performed under the assumption of autosomal-recessive inheritance, full penetrance, and a disease gene frequency of 0.0001. Multipoint LOD scores were calculated with the program ALLEGRO29 and presented graphically with Homozygosity-Mapper (http://www.homozygositymapper.org/).

### Human mutational analysis

Genomic DNA was isolated by standard methods directly from blood samples or from lymphocyte cultures after Epstein-Barr virus transformation. Amplification of genomic DNA was performed in a volume of 50 μl containing 30 ng DNA, 50 pmol of each primer, 2 mM dNTPs, and 1.0 U GoTaq DNA polymerase (Promega Corporation, #M3001) or 1.0 U MolTaq polymerase (Molzym Corporation, #P-016). PCR amplifications were carried out by an initial denaturation step at 94°C for 3 min, and 33 cycles as follows: 94°C for 30 sec, 58–60°C for 30 sec, and 72°C for 70 sec, with a final extension at 72°C for 10 min. PCR products were verified by agarose gel electrophoresis, purified and sequenced bi-directionally. Sequence data were evaluated using the CodonCode software.

Mutations in OI families were identified by Sanger sequencing, and sequencing primers for all exons analyzed are available on request.

### Sperm collection and analysis

An ejaculate from individual AL-III-9 was analyzed by light microscopy:

The spermiogram is summarized in S5 Table.

### Immunofluorescence analysis

Immunofluorescence analysis was performed as described [26]. Polyclonal rabbit anti-DNALI1, anti-DNAH5, anti-DNAI1 and monoclonal anti-DNAI2 were reported previously [35] as well as monoclonal anti-GAS8 [36]. Monoclonal mouse anti acetylated-α-tubulin (T7451) was obtained from Sigma (Germany). Polyclonal rabbit anti-MNS1 (HPA039975) and anti-CCDC114 (HPA042524) were obtained from Atlas Antibodies (Sweden). Anti-mouse Alexa Fluor 488 and anti-rabbit Alexa Fluor 546 were used as secondary antibodies (Molecular Probes, Invitrogen). DNA was stained with Hoechst 33342 (Sigma). Images were taken with a Zeiss Apotome Axiovert 200 and processed with AxioVision 4.8 and Adobe Creative Suite 4 software.

### Transmission electron microscopy (TEM)

TEM was performed as described [37].

### cDNA cloning

A Gateway NTAP-MNS1 construct used for tandem affinity purification was described [38]. MNS1 was subcloned into Gateway destination vectors for co-immunoprecipitation and yeast two hybrid analysis via LR Clonase reaction as described [39]. All MNS1 constructs were confirmed by sequencing and matched gene accession number: NM_018365.2. CCDC114 Gateway constructs used for interaction studies with MNS1 were described [29].

### Co-immunoprecipititation and Western Blotting (WB)

Co-IP and WB were performed as previously described [39]. Briefly, HEK293 cells were transfected with plasmids encoding myc- and FLAG-tagged cDNA constructs using Gene Juice (Novagen) at approximately 0.1 μg DNA per ml of media. Within 24 hrs, cells were collected in 1× PBS and lysed in 1 ml of the following buffer: 50 mM Tris-Cl, pH 8.0, 150 mM NaCl, 1% IGEPAL, 0.5 mM EDTA, and 10% glycerol supplemented with protease (Roche Complete) and phosphatase inhibitors (Cocktails 2 and 3, Sigma Aldrich). Lysates were centrifuged at 16,000 × g for 30 min. at 4°C. Approximately 2 mg of each lysate was precleared with 4 μg of rabbit control IgG antibody for 2 hrs. at 4°C, and then incubated with MagSi/protein A beads (MagnaMedics, Germany) for 1 hr. Lysates were then incubated with 4 μg of rabbit anti-FLAG or anti-myc antibody overnight at 4°C, and then incubated with MagSi/protein A beads for 1 hr. to capture immunoprecipitates. Bead complexes were washed four times in lysis buffer and then resuspended in 1× LDS buffer supplemented with DTT (1/8 lysis volume) and heated for 10 min. at 90°C. Lysates were electrophoresed in NuPAGE 4–12% Bis-Tris gels, transferred to PVDF filters, and subsequently immunoblotted with either anti-myc (A7) or anti-FLAG (M2) mouse monoclonal antibodies. PVDF filters were washed three times in TBS-T (10 minutes each) before blocking in 5% BSA for 2 hours at room temperature. Filters were then washed three times (10 minutes each) before incubation with primary antibody (diluted in TBS-T) overnight at 4°C. Filters were washed three times (10 minutes each) and then incubated with secondary antibody for 1 hour at room temperature. Filters were then washed four times and developed by ECL using Prime Western Blotting Detection Reagent (Amersham). Images were digitally acquired using a FUSION-SL Advance Imager (PeqLab) and modified for contrast using Adobe Photoshop v. CS4. All wash and incubation steps were performed with gentle shaking.

Western Blots in respiratory and sperm lysates were performed similarly but PVDF filters were blocked overnight with 5% milk at 4°C and incubated with primary antibody (diluted in 5% milk) for 3 hours.

The following antibodies were used: Rabbit polyclonal anti-MNS1 (HPA039975), rabbit polyclonal anti-CCDC114 (HPA042524) and rabbit polyclonal anti-myc (1:25; clone A-14, Santa Cruz).

### Yeast two-hybrid analysis

Binary interaction between CCDC114 and MNS1 was tested as described [39].

### Cloning and isolation of plasmid for *in situ* hybridization

Using primers Mns1-F: 5`-aagaagcgtgaggagatgga-3´ and Mns1-R: 5`cggccttgctatgaagactc-3´, a 728 bp fragment of *Mns1* (NM_008613.3) was amplified from mouse testis cDNA. The fragment was ligated into the pCRII-TOPO vector by TOPO cloning reaction (Invitrogen; Thermo Fisher Scientific) and the resulting plasmid subsequently transformed into chemically competent *E*.*coli* cells. Positive clones were selected for plasmid preparation (plasmid midi kit; Qiagen) and the isolated plasmid was checked for the correct insert by Sanger sequencing.

### Generation of *in situ* hybridization probes

NotI or KpnI (Fermentas; Thermo Fisher Scientific) digested plasmids served as templates for *in vitro* transcription of sense and antisense probes, respectively by T7 or SP6 polymerase incorporating Digoxigenin (DIG) -11-UTPs into the newly synthesized probes.

### Animal experiments

All experiments utilizing animals were performed under the approval by local government authorities (Landesamt für Natur, Umwelt und Verbraucherschutz Nordrhein-Westfalen, Germany (AZ 84–02.05.20.12.164, and AZ 84–02.05. 5.15. 012).

Wildtype CD-1 mice were mated in the evening and the females checked for vaginal plugs in the morning of the following day. Pregnant mice were sacrificed at day 7 after plug detection, corresponding to embryonic day 8.25 dpc (days post coitum) and embryos were dissected in ice cold 1xPBS. After fixation in 4%PFA in 1x PBS overnight, embryos were transferred to methanol and stored in methanol at -80°C until use.

### *In situ* hybridization

Mouse embryos were transferred to PBT (1xPBS + 0,1% Tween) and bleached in H_2_O_2_ in PBT for 1 hour. Digestion with proteinase K (10μg/ml) was performed and stopped after 8 minutes by the use of glycine (2mg/ml). After fixation with fixing solution (4%PFA and 0,2% Glutaraldehyde in PBT), embryos were rinsed and washed with PBT and preincubated with hybridization solution (50% formamide, 0,5% CHAPS, 1,3x SSC, 5 mM EDTA, 50μg/ml Yeast t-RNA; 700U/ml Heparin, 0,2%Tween) at 65°C for few hours. Hybridization with sense or antisense probes was performed in hybridization solution at 65°C overnight. The following day, after washing twice with hybridization solution and twice with MABT (Maleic acid (500mM), NaCl (750 mM), NaOH (1M), 0,2%Tween) embryos were blocked in blocking solution (2% Boehringer blocking reagent (Roche; Merck) in MABT) for 1 hour, and blocking solution with 20% lamp serum for additional 2–3 hours. Anti-DIG antibody was diluted 1:2000 in blocking solution with 20% lamp serum. Binding of the antibody was performed in blocking solution with 20% lamp serum at 4°C overnight. The following day, embryos were washed three times with MABT and twice with NTMT (NaCl (100mM), Tris (100mM), MgCl2 (50mM), Tween (0,2%)). Color development was performed utilizing NBT/ BCIP (Roche; Merck) in NTMT. After fixation in fixing solution and transfer to 80% glycerol in PBT, embryos were imaged utilizing a Nikon Digital Sight DS-L3 camera mounted on a Nikon SMZ1000 stereomicroscope and images were processed using creative suite (Adobe).

### Web resources

Aceview, https://www.ncbi.nlm.nih.gov/IEB/Research/Acembly/

Homozygosity-Mapper, http://www.homozygositymapper.org/

Exome Aggregation Consortium (ExAC), http://exac.broadinstitute.org/

Genome Aggregation Database (*gnomAD*), http://gnomad.broadinstitute.org/

Database of Genomic Variants, http://projects.tcag.ca/variation/

dbSNP, http://www.ncbi.nlm.nih.gov/SNP/

1000 Genomes Project human polymorphism database, http://www.1000genomes.org/

National Heart, Lung and Blood Institute–Exome Sequencing Project, http://evs.gs.washington.edu/EVS/

Online Mendelian Inheritance in Man, http://www.omim.org/

## Supporting information

S1 Fig Linkage analysis in individual OI-11 II6. (PDF)

S2 Fig DNAH5 localizes normally to the axonemes with MNS1 deficiency. (PDF)

S3 Fig *MNS1* mutations do not result in additional structural defects. (PDF)

S4 Fig ODA-DC associated protein ARMC4 localizes normally in MNS1-deficient cilia but fails to assemble in the distal axonemes in respiratory epithelial cells with a double deficiency of MNS1 and DNAH5. (PDF)

S1 Table Filtering process for gene variants identified through whole exome sequencing. (PDF)

S2 Table List of homozygous variants after filtering in individual AL-IV-3. (PDF)

S3 Table List of homozygous variants after filtering in individual BG-II-1. (PDF)

S4 Table List of homozygous variants after filtering in individual MS-II-1. (PDF)

S5 Table Spermiogram data. (PDF)
