# Supplementary material for: Homozygous loss-of-function mutations in MNS1 cause laterality defects and likely male infertility
Source: PLoS Genet. 2018 Aug 27;14(8):e1007602. doi: 10.1371/journal.pgen.1007602 (PMC6128653; doi:10.1371/journal.pgen.1007602)
Supplement: S1 Fig — (PDF) [file pgen.1007602.s001.pdf]

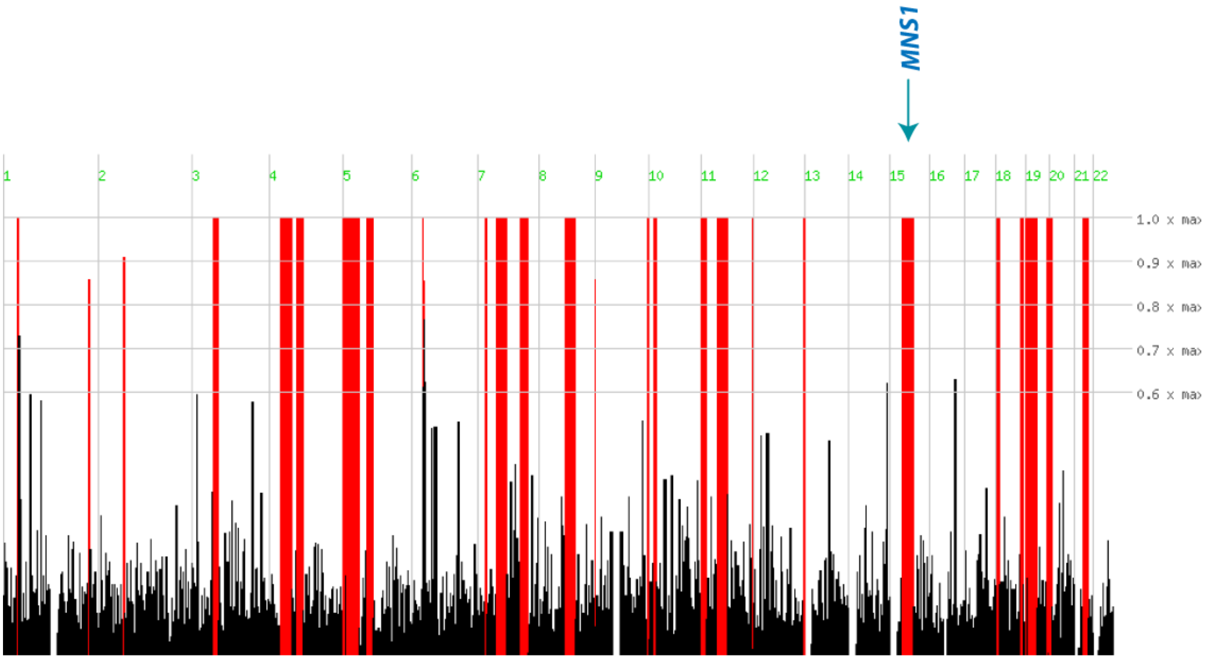

S1 Fig. Linkage analysis in individual OI-11 II6. Genome-wide homozygosity is displayed as bar charts. Candidate regions of homozygosity by descent with scores higher than 80% of the maximum score are displayed as red bars. *MNS1* is positioned within one of the maximum score peaks (arrow).
