# Supplementary material for: Homozygous loss-of-function mutations in MNS1 cause laterality defects and likely male infertility
Source: PLoS Genet. 2018 Aug 27;14(8):e1007602. doi: 10.1371/journal.pgen.1007602 (PMC6128653; doi:10.1371/journal.pgen.1007602)
Supplement: S2 Fig — (PDF) [file pgen.1007602.s002.pdf]

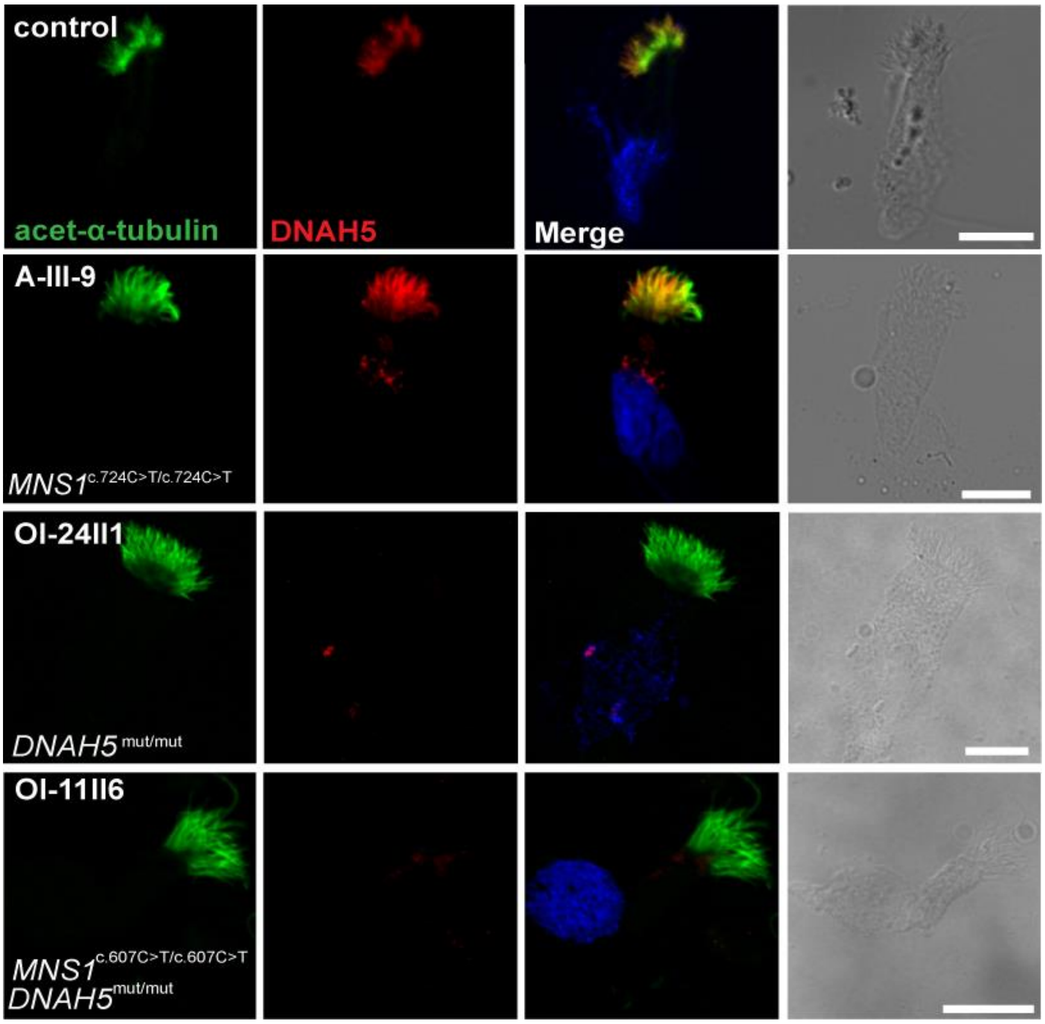

S2 Fig. DNAH5 localizes normally to the axonemes with MNS1 deficiency.

Respiratory epithelial cells from control and affected individuals: AL-III-9 carrying bi-allelic *MNS1* mutations, OI-11 II6 carrying bi-allelic *MNS1* and *DNAH5* mutations and OI-24 II1 carrying no mutations in *MNS1* but identical bi-allelic *DNAH5* mutations as OI-11 II6. For space issues, OI-24 II1 is described as *DNAH5*$^{mut/mut}$ instead of *DNAH5*$^{c.13432\_13435delCACT/\ c.13432\_13435delCACT}$. Cells were double-labeled with antibodies directed against acetylated alpha-tubulin (green) and DNAH5 (red). Nuclei were stained with Hoechst 33342 (blue). Both proteins co-localize (yellow) along the ciliary axonemes in cells from the unaffected controls and cells from AL-III-9, while in respiratory cells of OI-11 II6 and OI-24 II1, DNAH5 is undetectable consistent with a defect of the outer dynein arms.
