# Supplementary material for: Homozygous loss-of-function mutations in MNS1 cause laterality defects and likely male infertility
Source: PLoS Genet. 2018 Aug 27;14(8):e1007602. doi: 10.1371/journal.pgen.1007602 (PMC6128653; doi:10.1371/journal.pgen.1007602)
Supplement: S3 Fig — (PDF) [file pgen.1007602.s003.pdf]

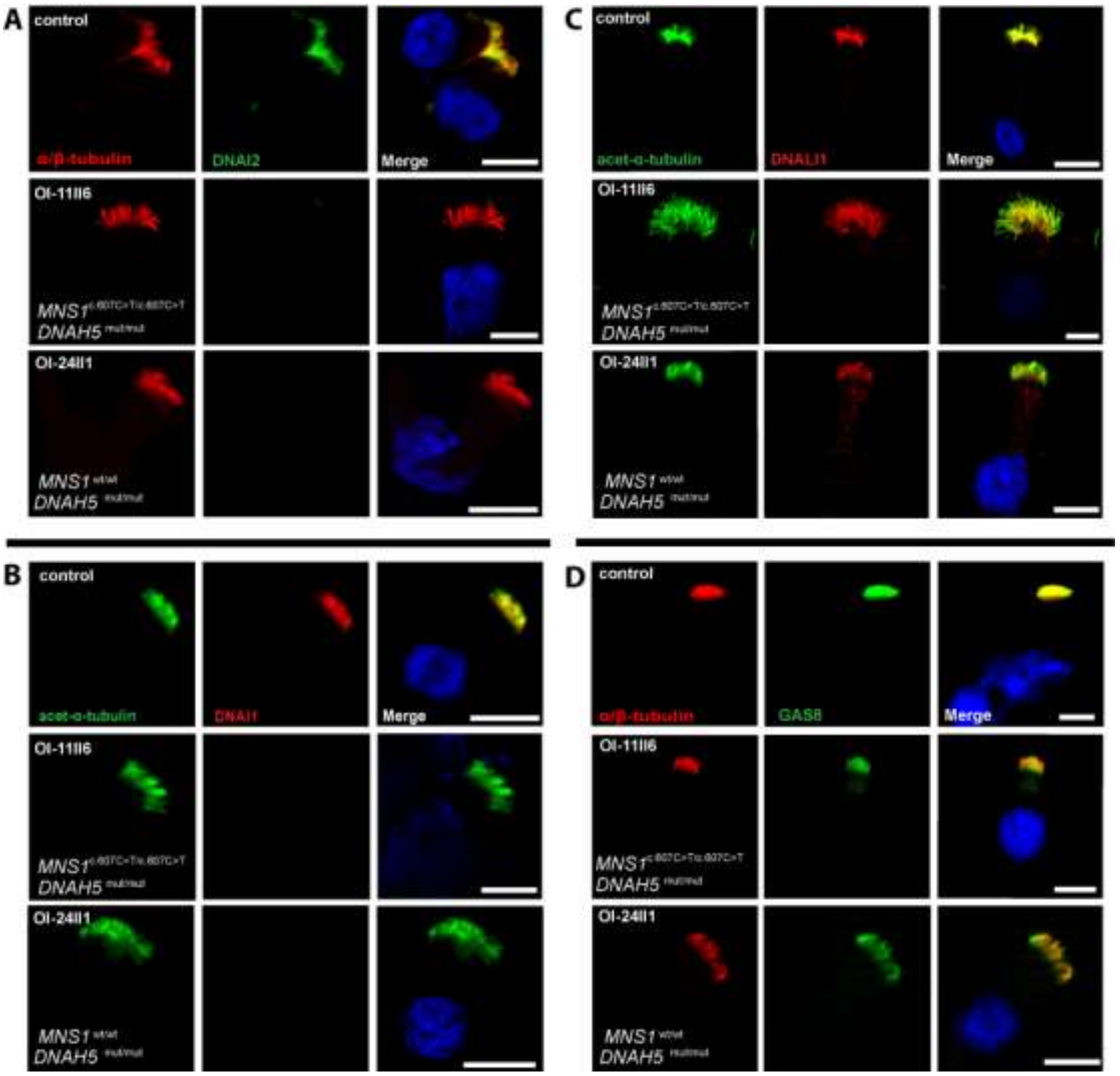

S3 Fig. *MNS1* mutations do not result in additional structural defects.

A-D. Respiratory epithelial cells from control and PCD-affected individuals: OI-11 II6 carrying bi-allelic *MNS1* and *DNAH5* mutations and OI-24 II1 carrying no mutations in *MNS1* but identical bi-allelic *DNAH5* mutations as OI-11 II6. **A**. Cells were double-labeled with antibodies directed against alpha-beta tubulin (red) and DNAI2 (green). **B**. Cells were double-labeled with antibodies directed against acetylated alpha-tubulin (green) and DNAI1 (red). **A-B** DNAI2 and DNAI1 localize (yellow) along the cilia in cells from the unaffected controls. In contrast, in OI-11 II6 and OI-24 II1 cells, neither DNAI2 (**A**) nor DNAI1 (**B**) are detectable in the ciliary axonemes suggesting that MNS1 and DNAH5 deficiency leads to ODA defects. **C**. Cells were double-labeled with antibodies directed against acetylated alpha-tubulin (green) and DNALI1 (red). **D**. Cells were double-labeled with antibodies directed against alpha-beta tubulin (red) and GAS8 (green). **C-D** DNALI1 and GAS8 localize (yellow) along the cilia in cells from the unaffected control and both PCD-affected individuals OI-11 II6 and OI-24 II1, indicating that neither *MNS1* nor *DNAH5* mutations result in defects of the inner dynein arms nor in defects of the nexin dynein regulatory complexes. Nuclei were stained with Hoechst 33342 (blue). Scale bars, 10µm.
