# Supplementary material for: Homozygous loss-of-function mutations in MNS1 cause laterality defects and likely male infertility
Source: PLoS Genet. 2018 Aug 27;14(8):e1007602. doi: 10.1371/journal.pgen.1007602 (PMC6128653; doi:10.1371/journal.pgen.1007602)
Supplement: S4 Fig — (PDF) [file pgen.1007602.s004.pdf]

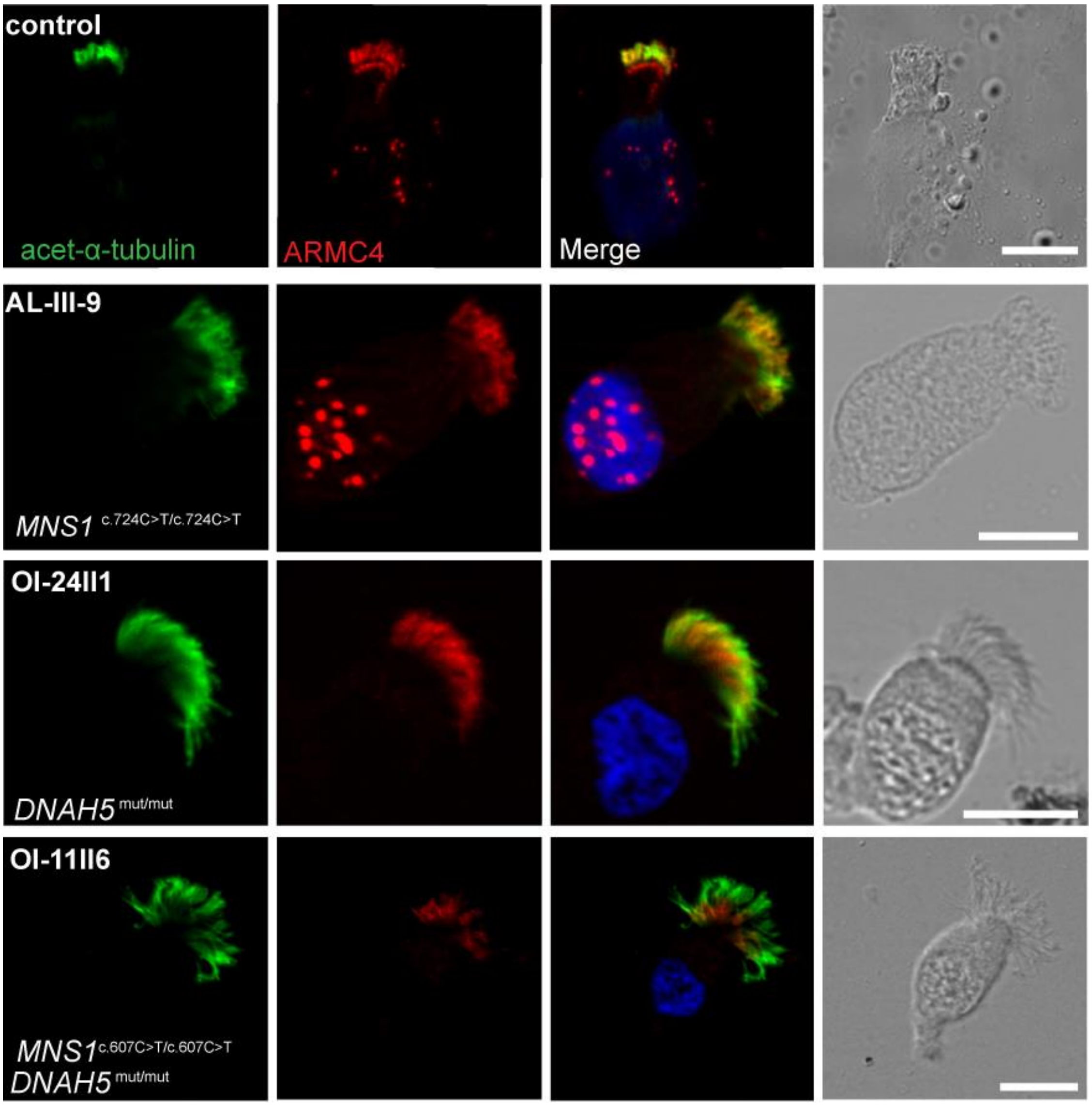

S4 Fig. ODA-DC associated protein ARMC4 localizes normally in MNS1-deficient cilia but fails to assemble in the distal axonemes in respiratory epithelial cells with a double deficiency of MNS1 and DNAH5.

Respiratory epithelial cells from control and affected individuals: AL-III-9 carrying bi-allelic *MNS1* mutations, OI-11 II6 carrying bi-allelic *MNS1* and *DNAH5* mutations and OI-24 II1 carrying no mutations in *MNS1* but identical bi-allelic *DNAH5* mutations as OI-11 II6. For space issues, OI-24 II1 is described as $DNAH5^{mut/mut}$ instead of $DNAH5^{c.13432\_13435delCACT/\ c.13432\_13435delCACT}$. Cells were double-labeled with antibodies directed against acetylated alpha-tubulin (green) and ARMC4 (HPA037829, Atlas antibodies) (red). Nuclei were stained with Hoechst 33342 (blue). Both proteins co-localize (yellow) along the ciliary axonemes in cells from the unaffected controls, AL-III-9 and OI-24 II1, while in cells of OI-11 II6, ARMC4 localizes only to the proximal part of the ciliary axonemes, indicating that recessive loss-of-function mutations in *MNS1* when combined with loss-of-function mutations in *DNAH5* might affect the distal localization of ODA-DC associated proteins and might play a role in docking or anchoring the ODA subunits or in regulating this process. Scale bars, 10μm.
