# Supplementary material for: Homozygous loss-of-function mutations in MNS1 cause laterality defects and likely male infertility
Source: PLoS Genet. 2018 Aug 27;14(8):e1007602. doi: 10.1371/journal.pgen.1007602 (PMC6128653; doi:10.1371/journal.pgen.1007602)
Supplement: S1 Table — (PDF) [file pgen.1007602.s005.pdf]

S1 Table. Filtering process for gene variants identified through whole exome sequencing

	MS-II-1	AL-IV-3	BG-II-1
Total number of homozygous coding variants or within 20 bp of intron-exon boundaries with MAF <0.5% in Exac, 1000 genomes and eps6500	44 variants (see Table S4 for details)	26 variants (see Table S2 for details)	23 variants (see Table S3 for details)
Of which stop, frameshift or consensus splice variants	AGAP3: rs766984834, c.94_95insGGGG:p.C32fs<br>MNS1: rs185005213, MNS1:NM_018365:c.C724T:p.R242X<br>MST1L: rs200532237, NM_001271733:c.811dupG:p.A271fs<br>SLAIN1: rs201380414, NM_001242868:c.219_220insGG:p.A73fs	MNS1: rs185005213, MNS1:NM_018365:p.R242X	MNS1: rs185005213, MNS1:NM_018365:p.R242X<br>MYO3A:NM_017433:p.T26fs<br>VEZF1:NM_007146:p.Q349QQQ<br>UBXN11: NM_001077262.1:p.GPGPSPCPG506G
Of which (associated with left-right body axis establishment in the literature) segregated with the phenotype in the family	MNS1: rs185005213, MNS1:NM_018365:c.C724T:p.R242X	MNS1: rs185005213, MNS1:NM_018365:p.R242X	MNS1: rs185005213, MNS1:NM_018365:p.R242X

MAF: Minor allele frequency
