# Supplementary material for: Homozygous loss-of-function mutations in MNS1 cause laterality defects and likely male infertility
Source: PLoS Genet. 2018 Aug 27;14(8):e1007602. doi: 10.1371/journal.pgen.1007602 (PMC6128653; doi:10.1371/journal.pgen.1007602)
Supplement: S2 Table — (PDF) [file pgen.1007602.s006.pdf]

S2 Table. List of homozygous variants left after filtering in individual AL-IV-3

>chromoso	chrStart	refSeq	varSeq	varType	rsidList	genesList	protImpactList	aaChange	globalAlleleFreq
12	475269	A	T	SNP (x2)		KDM5A	MISSENSE	I-123-N	0
11	65307193	G	A	SNP (x2)		LTBP3	MISSENSE	A-1182-V	0
3	1418745	G	A	SNP (x2)	rs140014929	CNTN6	MISSENSE	V-718-I	0,01
8	98991129	C	T	SNP (x2)	rs146982976	MATN2	MISSENSE	A-325-V	0,26
14	20849189	G	C	SNP (x2)	rs144150517	TEP1	MISSENSE	R-1555-G	0,17
3	50614965	G	A	SNP (x2)	rs2232250	HEMK1	MISSENSE	R-192-Q	0,33
8	99042725	A	C	SNP (x2)		MATN2	MISSENSE	I-798-L	0
3	1,68E+08	G	A	SNP (x2)	rs61750375	SERPINI1	MISSENSE	V-97-I	0,46
1	2,47E+08	G	A	SNP (x2)	rs567370905	CNST	MISSENSE	S-113-N	0,12
1	2,03E+08	C	T	SNP (x2)		PPFIA4	MISSENSE	P-528-L	0
1	10529326	A	G	SNP (x2)	rs138842024	DFFA	MISSENSE	I-69-T	0,34
12	863309	C	A	SNP (x2)	rs72647372	WNK1	MISSENSE	P-193-Q	0,05
22	38318211	G	C	SNP (x2)		MICALL1	MISSENSE	D-268-H	0
1	10714097	C	T	SNP (x2)	rs143974084	CASZ1	MISSENSE	A-673-T	0,01
3	50329885	A	T	SNP (x2)	rs200409823	IFRD2	MISSENSE	S-5-T	0,03
1	12337667	C	T	SNP (x2)	rs12407578	VPS13D	MISSENSE	S-1341-L	0,27
3	48310010	G	A	SNP (x2)		ZNF589	MISSENSE	V-277-I	0
3	50379289	T	C	SNP (x2)	rs114329675	ZMYND10	MISSENSE	Q-358-R	0,17
3	44285446	A	G	SNP (x2)	rs17076541	TOPAZ1	MISSENSE	Q-483-R	0,35
12	5154242	C	T	SNP (x2)	rs17215402	KCNA5	MISSENSE	P-310-L	0,37
12	1023269	G	C	SNP (x2)		RAD52	MISSENSE	S-329-C	0
11	63992155	G	A	SNP (x2)	rs143200811	TRPT1	MISSENSE	P-123-L	0,03
12	51740410	A	G	SNP (x2)	rs116944010	CELA1	MISSENSE	Y-5-H	0,02
12	51740409	T	G	SNP (x2)	rs117443541	CELA1	MISSENSE	Y-5-S	0,02
11	62294549	T	G	SNP (x2)	rs200444167	AHNAK	MISSENSE	H-2447-P	0,01
15	56736015	G	A	SNP (x2)	rs185005213	MNS1	NONSENSE	R-242-*	0,04
