# Supplementary material for: Homozygous loss-of-function mutations in MNS1 cause laterality defects and likely male infertility
Source: PLoS Genet. 2018 Aug 27;14(8):e1007602. doi: 10.1371/journal.pgen.1007602 (PMC6128653; doi:10.1371/journal.pgen.1007602)
Supplement: S3 Table — (PDF) [file pgen.1007602.s007.pdf]

S3 Table. List of homozygous variants left after filtering in individual BG-II-1

>chromoso	chrStart	refSeq	varSeq	varType	rsidList	genesList	protImpactList	aaChange	globalAlleleFreq
10	26241115	AC	TA	SNP (x2)		MYO3A	NONSENSE	T-26-X	
17	56056605	-	TGCTGC	INS (x2)		VEZF1	INSERT+	Q-349-QQQ	0
1	26608812	CCAGGACA	-	DEL (x2)		UBXN11	DELETE+	GPGPSPCPG-506-G	0
5	40955530	G	C	SNP (x2)	rs121964921	C7	MISSENSE	G-379-R	0,01
18	33935547	G	A	SNP (x2)		FHOD3	MISSENSE	D-71-N	0
15	93588394	T	A	SNP (x2)		RGMA	MISSENSE	Y-404-F	0
10	49654502	T	C	SNP (x2)	rs148992704	ARHGAP22	MISSENSE	M-693-V	0,01
17	33977795	A	G	SNP (x2)		AP2B1	MISSENSE	I-595-V	0,01
15	44856764	A	G	SNP (x2)	rs150571352	SPG11	MISSENSE	F-2378-L	0,02
X	69459599	C	T	SNP (x2)	rs141044914	AWAT1	MISSENSE	P-216-L	0,05
17	66364750	G	A	SNP (x2)	rs144631983	ARSG	MISSENSE	V-256-M	0,03
8	110460501	T	A	SNP (x2)		PKHD1L1	MISSENSE	V-1969-E	0
X	30236905	G	A	SNP (x2)	rs183272477	MAGEB2	MISSENSE	A-70-T	0,06
X	118222854	G	A	SNP (x2)	rs376769242	KIAA1210	MISSENSE	S-780-F	0,01
X	34149690	G	A	SNP (x2)	rs367563899	FAM47A	MISSENSE	R-236-C	0,02
X	48780985	T	C	SNP (x2)		OTUD5	MISSENSE	T-507-A	0
10	51585255	A	C	SNP (x2)		NCOA4	MISSENSE	K-468-Q	0
5	35876524	C	G	SNP (x2)	rs148931962	IL7R	MISSENSE	T-439-S	0,04
X	83128603	C	G	SNP (x2)	rs150275297	CYLC1	MISSENSE	S-296-C	0,04
20	1610949	G	T	SNP (x2)		SIRPG	MISSENSE	P-362-Q	0
X	54783884	C	T	SNP (x2)		ITIH6	MISSENSE	E-875-K	0,01
15	56736015	G	A	SNP (x2)	rs185005213	MNS1	NONSENSE	R-242-*	0,04
