# Supplementary material for: Homozygous loss-of-function mutations in MNS1 cause laterality defects and likely male infertility
Source: PLoS Genet. 2018 Aug 27;14(8):e1007602. doi: 10.1371/journal.pgen.1007602 (PMC6128653; doi:10.1371/journal.pgen.1007602)
Supplement: S4 Table — (PDF) [file pgen.1007602.s008.pdf]

S4 Table. Homozygous variants after filtering in individual MS-II-1

CHROM	POS	ID	REF	ALT	GeneName	Func	ExonicFunc	AAChange	1000g2015aug_a	esp6500siv	ExAC_ALL
5	7757704	rs764722596	G	GCCCCAGAGC	ADCY2	splicing	.	.	.	.	8.33E-03
7	150783911	rs769408537	A	G	AGAP3	exonic	missense SNV	c.A83G:p.Q28R	.	.	0
7	150783917	rs775200524	T	G	AGAP3	exonic	missense SNV	c.T89G:p.L30R	.	.	0
7	150783922	rs766984834	T	TGGGG	AGAP3	exonic	frameshift INS	c.94_95insGGGG:p.C32fs	.	.	0
7	150783919	rs776100575	GTCT	G	AGAP3	exonic	nonframeshift DEL	c.92_94del:p.31_32del	.	.	0
X	48925533	.	G	A	CCDC120	exonic	missense SNV	c.G1883A:p.R628H	0.000264901	.	0.0030
14	39784012	rs554677567	A	ATGTGTGTGT	CTAGE5	splicing	.	.	0.00479233	.	.
15	45401064	.	AGCTGGGCA	A	DUOX2	exonic	nonframeshift DEL	c.1300_1320del:p.434_440del	.	.	.
8	11666218	rs71711801	GTCCCAC	G	FDFT1	exonic	nonframeshift DEL	c.193_198del:p.65_66del	.	.	.
6	32632646	rs9274387	G	T	HLA-DQB1	exonic	missense SNV	c.C308A:p.T103N	.	.	0.0036
19	55239223	.	G	A	KIR3DL3	exonic	missense SNV	c.G502A:p.V168I	.	.	.
15	43621201	rs774895599	A	C	LCMT2	exonic	missense SNV	c.T1487G:p.F496C	.	.	1.65E-02
21	35334666	rs766424	C	T	LINC00649	exonic	missense SNV	c.C377T:p.S126F	.	.	.
3	100170600	rs71132521	A	ATCCTAGAAG	LNP1	exonic	nonframeshift INS	[1]see below	.	.	.
15	56736015	rs185005213	G	A	MNS1	exonic	stopgain	c.C724T:p.R242X	0.000399361	7.7e-05	0.0004
1	17086085	rs200532237	G	GC	MST1L	exonic	frameshift INS	c.811dupG:p.A271fs	.	.	0.0010
X	153421854	.	A	T	OPN1LW	exonic	missense SNV	c.A830T:p.Y277F	.	.	.
X	153421859	.	G	T	OPN1LW	exonic	missense SNV	c.G835T:p.V279F	.	0.0002	1.15E-02
X	153421844	.	A	G	OPN1LW	exonic	missense SNV	c.A820G:p.I274V	.	.	.
X	153421847	.	T	C	OPN1LW	exonic	missense SNV	c.T823C:p.F275L	.	.	.
X	153421849	.	T	G	OPN1LW	exonic	missense SNV	c.T825G:p.F275L	.	.	1.15E-02
X	153496198	.	T	A	OPN1MW2	exonic	missense SNV	c.T926A:p.F309Y	.	.	0.0001
X	153496164	.	C	G	OPN1MW2	exonic	missense SNV	c.C892G:p.P298A	0.0015894	.	0.0003
X	153496125	.	G	A	OPN1MW2	exonic	missense SNV	c.G853A:p.A285T	.	.	0.0005
3	16926607	rs7626448	T	C	PLCL2	exonic	unknown	UNKNOWN	.	.	0
3	16926544	rs368284573	T	G	PLCL2	exonic;splici	unknown	UNKNOWN	.	.	0
X	153036027	.	C	T	PLXNB3	exonic	missense SNV	c.C1940T:p.P647L	.	.	6.37E-02
21	14982886	rs142207431	G	A	POTED	exonic	missense SNV	c.G337A:p.G113S	.	.	.
21	14982952	rs117112505	A	G	POTED	exonic	missense SNV	c.A403G:p.I135V	.	.	.
21	15013735	rs78643169	A	G	POTED	exonic	missense SNV	c.A1603G:p.M535V	.	.	.
1	33430102	rs113840389	T	G	RNF19B	exonic	missense SNV	c.A185C:p.Q62P	.	.	.
8	10467637	rs369606728	T	TCCTCTAACTG	RP1L1	exonic	nonframeshift INS	[2]see below	.	.	.
13	78272267	rs201380414	T	TGG	SLAIN1	exonic	frameshift INS	c.219_220insGG:p.A73fs	.	.	0
3	14444329	rs67559364	CT	C	SLC6A6	splicing	.	.	.	.	.
15	51041909	rs145832874	C	T	SPPL2A	exonic	missense SNV	c.G101A:p.G34E	0.00259585	0.0011	0.0019
3	12046256	rs782385358	GCCCCCCCCC	G	SYN2	exonic	unknown	UNKNOWN	.	.	0
3	12046268	rs782162668	GCGCGA	G	SYN2	exonic	unknown	UNKNOWN	.	.	0
3	12046222	rs71624921	A	C	SYN2	exonic	unknown	UNKNOWN	.	.	0
3	12046215	rs2623876	G	C	SYN2	exonic	unknown	UNKNOWN	.	.	0
2	46707808	rs70940616	C	CAGCGGCAGC	TMEM247	exonic	nonframeshift INS	[3]see below	.	.	.
3	42251577	rs10634555	C	CGGA	TRAK1	exonic	nonframeshift INS	[4]see below	.	.	.
14	38183928	rs78042024	G	A	TTC6	exonic	missense SNV	c.G1688A:p.G563E	0.00439297	.	0.0028
4	9236859	rs776377841	A	T	USP17L15	exonic	missense SNV	c.A749T:p.N250I	.	.	0

[1]c.194_195insTCCTAGAAGGCATTCTCATGAGGACCAGGAATTCCGATGCCGATCGTCTGACCGTCT:p.H65delinsHPRRHSHEDQEFRCRSSDRL

[2]c.3970_3971insGGACTAAAGTAATAGAAGGGCTGCAAGAAGAGAGGGTGCAGTTAGAGG:p.E1324delinsGTKVIEGLQEERVQLEE

[3]c.382_383insAGCGGCAGCACGAGGTGGTGATGGAGCAGCTGCAGCGGG:p.Q128delinsQRQHEVVMEQLQRE

[4]c.1841_1842insGGA:p.T614delinsTE
