# Supplementary material for: Homozygous loss-of-function mutations in MNS1 cause laterality defects and likely male infertility
Source: PLoS Genet. 2018 Aug 27;14(8):e1007602. doi: 10.1371/journal.pgen.1007602 (PMC6128653; doi:10.1371/journal.pgen.1007602)
Supplement: S5 Table — (PDF) [file pgen.1007602.s009.pdf]

S5 Table. Spermiogram data

**Individual**	**Volume** (≥ 1.5 ml)	**Color**[1]	**Viscosity**[2]	**Concentration** (≥ 15 x 10^6 cells/ ml)	**Motility**[3] **(**≥ 32% progressive motile (PR) sperm cells*)	**Morphology** (≥ 4% normal formed sperm cells*)
A-III-9	3 ml	normal	normal	22 x 10^6 cells/ ml	PR: 07%	< 1%
					NP: 05%	
					IM: 88%	
B-II-1	3 ml	creamy	normal	80 x 10^6 cells/ ml	PR: 20%	10%
					NP: N.A.	
					IM: N.A.	

n.a.: not available

Reference: WHO laboratory manual for the examination and processing of human semen. 5th ed. World Health Organization, Geneva, 2010.

[1] According to latest WHO guidelines (2010) a normal liquefied semen sample has a homogeneous, grey-opalescent appearance. It may appear less opaque if the sperm concentration is very low; the colour may also be different, i.e. red-brown when red blood cells are present (haemospermia), or yellow in a man with jaundice or taking certain vitamins or drugs.

[2] A semen sample with normal viscosity leaves the pipette in small discrete drops. If viscosity is abnormal, the drop will form a thread more than 2 cm long.

[3] Sperm motility according to latest WHO guidelines (2010) is assessed by categorizing sperm cells in the analyzed ejaculate in progressive motile (PR), non-progressive motile (NP) and immotile sperm cells. The respective percentages have to be determined.

*32% for motility and 4% for morphology are the lower limits for normality.
